# Genes to predict VO_2max_ trainability: a systematic review

**DOI:** 10.1186/s12864-017-4192-6

**Published:** 2017-11-14

**Authors:** Camilla J. Williams, Mark G. Williams, Nir Eynon, Kevin J. Ashton, Jonathan P. Little, Ulrik Wisloff, Jeff S. Coombes

**Affiliations:** 1 Centre for Research on Exercise, Physical Activity and Health (CRExPAH), School of Human Movement and Nutrition Sciences, The University of Queensland, Brisbane, Queensland Australia; 2 Molecular Genetics Department, Mater Pathology, South Brisbane, Queensland Australia; 3 Institute of Sport, Exercise and Active Living (ISEAL), Victoria University, Melbourne, 8001 Australia; 4 Faculty of Health Sciences and Medicine, Bond University, Robina, Queensland Australia; 5 School of Health and Exercise Sciences, University of British Columbia, Okanagan, Canada; 6 Cardiac K.G. Jebsen Center for Exercise in Medicine at Department of Circulation and Medical Imaging, Norwegian University of Science and Technology, Trondheim, Norway

**Keywords:** Cardiorespiratory fitness, VO_2max_, Predictor genes, Training

## Abstract

**Background:**

Cardiorespiratory fitness (VO_2max_) is an excellent predictor of chronic disease morbidity and mortality risk. Guidelines recommend individuals undertake exercise training to improve VO_2max_ for chronic disease reduction. However, there are large inter-individual differences between exercise training responses. This systematic review is aimed at identifying genetic variants that are associated with VO_2max_ trainability.

**Methods:**

Peer-reviewed research papers published up until October 2016 from four databases were examined. Articles were included if they examined genetic variants, incorporated a supervised aerobic exercise intervention; and measured VO_2max_/VO_2peak_ pre and post-intervention.

**Results:**

Thirty-five articles describing 15 cohorts met the criteria for inclusion. The majority of studies used a cross-sectional retrospective design. Thirty-two studies researched candidate genes, two used Genome-Wide Association Studies (GWAS), and one examined mRNA gene expression data, in addition to a GWAS. Across these studies, 97 genes to predict VO_2max_ trainability were identified. Studies found phenotype to be dependent on several of these genotypes/variants, with higher responders to exercise training having more positive response alleles than lower responders (greater gene predictor score). Only 13 genetic variants were reproduced by more than two authors. Several other limitations were noted throughout these studies, including the robustness of significance for identified variants, small sample sizes, limited cohorts focused primarily on Caucasian populations, and minimal baseline data. These factors, along with differences in exercise training programs, diet and other environmental gene expression mediators, likely influence the ideal traits for VO_2max_ trainability.

**Conclusion:**

Ninety-seven genes have been identified as possible predictors of VO_2max_ trainability. To verify the strength of these findings and to identify if there are more genetic variants and/or mediators, further tightly-controlled studies that measure a range of biomarkers across ethnicities are required.

## Background

The worldwide prevalence of chronic diseases, such as cardiovascular disease, cancers, stroke and diabetes is rising [1]. Low cardiorespiratory fitness is strongly associated with chronic diseases and premature mortality [2–7]. To alleviate the health and economic burden associated with low cardiorespiratory fitness, health guidelines across the world recommend individuals undertake regular exercise [1].

Exercise training can increase cardiorespiratory fitness and decrease chronic disease via a number of mechanisms [7]. Adaptations include improvements to cardiac size, stroke volume (increase in volume of blood pumped from the left ventricle), cardiac output (volume of blood pumped from the heart per minute), pulmonary blood flow and respiratory function, supply of oxygen-rich blood to working muscles (increased number of capillaries and blood volume), muscle mitochondrial function and content, oxidative enzyme capacity, vascular wall health and function, and biomechanical efficiency [2, 7]. It has been suggested that improvements in cardiorespiratory fitness in response to exercise training varies greatly between individuals, with some people responding well or very well (‘responders’ or ‘high-responders’) to exercise training, whereas others only have mild increases in their cardiorespiratory fitness following similar exercise training (‘low-responders’) [4, 5, 8–11]. Importantly, these responses need to be compared to within-subject random variation to ascertain true inter-individual differences [12]. The ability to change cardiorespiratory fitness is a multifactorial trait influenced by environmental factors (such as exercise training) and genetic factors [4, 5, 11]. Considering cardiorespiratory fitness is one of the best integrative predictors of morbidity and mortality risk, it may be important to understand how genetics predict the variability in response to exercise training. This knowledge could lead to targeted personalised exercise therapy to decrease the burden of chronic disease.

The gold standard measure for cardiorespiratory fitness is maximal oxygen uptake (VO_2max_), which is quantified as the maximal amount of oxygen the body can use in 1 min, during dynamic work with large muscle mass [13]. Research into human variation of VO_2max_ was first undertaken over forty years ago, with several authors identifying a strong genetic influence on VO_2max_ in twins [14, 15]. Subsequent studies have identified significant familial aggregation for VO_2max_ trainability. For example, authors have found greater variance between pairs of monozygotic (MZ; identical) twins than within pairs of twins for VO_2max_ training response after standardized aerobic training interventions [16, 17]. The strongest evidence to date on this topic was found in the HEalth, Risk factors, exercise training And GEnetics (HERITAGE) family study [18]. Four hundred seventy-three Caucasian adults from 99 nuclear families completed 20 weeks of Moderate Intensity Continuous Training (MICT). The average increase in VO_2max_ was 400 mL O_2_/min, with a range from − 114 to + 1097 mL/min. This difference was two and half times greater between families than within families, with a 47% heritability estimate for VO_2max_ training response [18]. A major limitation from these findings, however, is there was no comparator control group.

Since this familial longitudinal research, the Human Genome Project completed sequencing of the human genome resulting in significant advancements in genetic analysis capabilities. This led to a better understanding of genetic variations of large populations. Analyzing genetic variants on a population level using techniques such as candidate gene analysis, GWAS, whole genome and exome sequencing and RNA expression analysis (RNA-seq, or microarrays) has resulted in the possibility of developing ‘personalized genomics’. This aims for biological profiling to provide more effective health management and treatment [5]. However, research in the field of exercise genomics it still in its infancy and much work is needed before genomic tools could be utilized to personalize exercise training programs [19].

The aim of this study was to systematically review the literature and identify genetic variants that have been associated with VO_2max_ trainability following an aerobic exercise training intervention. Given the infancy of this research field, results should only be used to provide the basis for future research. This research should aim to confirm previous findings and investigate mediators that can influence gene expression. Importantly, future genetic studies in this area should attempt to investigate the physiological functions that contribute to improving VO_2max_ training response and overall health outcomes. Findings from ongoing research may assist clinical professionals to provide personalized evidenced-based medicine centered on phenotype, contributing to the fight against chronic disease.

## Methods

A comprehensive search of four databases (PubMed, Embase, Cinahl, Cochrane) was completed from their inception until October 2016. Studies focusing on genes and their VO_2max_/VO_2peak_ response to supervised aerobic training were sought with the following search terms: genetic profiling, polymorphism, single nucleotide polymorphisms, SNPs, genetic variants, predictor genes, trainability, endurance training, cardiovascular fitness, cardiorespiratory fitness, VO_2max_, VO_2peak_, aerobic power, aerobic fitness, aerobic capacity. A full list of search terms can be found at the end of this review.

Two authors (CW and JC) agreed on the criteria for inclusion. Articles were incorporated if they were: original, peer-reviewed research; included an aerobic intervention, with minimum 75% supervision; included genetic variant testing; included a maximal VO_2max_/_peak_ using direct gas analysis from an incremental test (pre and post intervention); conducted on humans; and written in English.

Using an extraction grid, one author (CW) conducted the initial screening analysis. After removing duplicates and scanning the titles and abstract of articles, those meeting the inclusion criteria were reviewed. Data recorded from the review consisted of the author’s name and place of study, study design, study sample, tissue source, genotyping method used, gene and variant examined, genotype, gene expression (if examined), intervention used, possible mediators (such as medications and health concerns), and the influence of the genetic variant investigated on VO_2max_ change. Further articles were retrieved from snowballing included articles from their reference lists. Articles included in the review are in Table 1.

**Table 1:** Summary of included articles

Author, Year, Country	Gene/s tested for VO_2_max trainability	Study Design	Study Sample	Tissue source	Method for Genotyping	Intervention
Xu, 2015, China	*ALAS2*	Single group, longitudinal. VO_2max_ and venous blood samples taken pre & post intervention.	*N* = 244 healthy Chinese males; 18-22 years (20 ± 1.76); wt 65.06 ± 9.59 kg; ht. 174.37 ± 6.16 cm. *N* = 72 randomly selected for HiHiLo training (69.8 ± 7.8 kg and 177.93 ± 5.26 cm).	Peripheral blood leucocytes	PCR protocol + separation on polyacrylamide gel	4 weeks; supervised HiLo training in hypoxia-training centre. Hi = bicycle ergometer for 30 mins at 75% VO_2max_, in 15.4% O_2_ concentrated environment, 3×/week for 4 weeks. Lo = same training but at lower elevation.
Yu, 2014, China	*APOE*	Single group, longitudinal. VO_2max,_ anthropometric and serum levels tested pre & post intervention.	*N* = 360; 180 Chinese males and females; age 32.8 ± 11.9 yrs.; BMI 25.4 ± 5.6 kg/m^2^ M; BMI 26 ± 6.2 kg/m^2^ F; no health concerns; inactive.	Peripheral blood leucocytes	PCR-(polymerase chain reaction)-RFLP (restriction fragment length polymorphism) assay	6 mths; progressive; supervised aerobic training; 60–85% VO_2max_.
Zarebska, 2014, Poland	*GSTP1*	Single group, longitudinal. VO_2max_, HR_max_, VE_max_, AT and body composition tested pre & post intervention; balanced diet prior to intervention (2000 kcal)	*N* = 66 Polish females; 19–24 yrs.; BMI 21.8 ± 2.1 kg/m^2^; no health concerns; inactive; no supplements or medications; non-smokers.	Buccal cells	TaqMan allelic discrimination assay using qPCR	3 mths; supervised; progressive MICT; 3×/wk.; 50–75% HR_max_; 30–60 min.
Ghosh, 2013, Singapore	*GWAS*	Retrospective, single-group longitudinal. V0_2max_ tested pre & post intervention.	HERITAGE WHITES: *n* = 473 Caucasians; 230 male & 243 females; no major health concerns; inactive.	Lymphoblastoid cell lines	Illumina Human CNV370-Quad Bead Chips	HERITAGE: 20 wks; supervised; progressive MICT; 3×/wk.; 55–75% VO_2max_; 30–50 min.
Bouchard, 2011, USA	*GWAS*	Retrospective HERITAGE: Single group, longitudinal; VO_2max_ tested pre & post intervention. DREW: RCT; VO_2max_ tested pre & post intervention. STRRIDE 1 & 2: RCT; VO_2max_ tested pre & post intervention.	HERITAGE WHITES: n = 473 Caucasians (252 women); 17–65 yrs.; inactive; no major health concerns HERITAGE BLACKS: *n* = 259 (177 women); 17–65 years; inactive; no major health concerns HERITAGE average age = 35.7 ± 14.5 yrs., BMI 25.8 ± 4.9 kg/m^2^. DREW study: *n* = 464 overweight or obese postmenopausal women; inactive; no major health concerns. STRRIDE 1 study: M&F; 40–65 yrs.; inactive; overweight, dyslipidemic and postmenopausal (F). STRRIDE 2 study: 18–70 yrs.; inactive; overweight, dyslipidemic. *N* = 183 for STRRIDE 1&2 studies.	Lymphoblastoid cell lines	Illumina Human CNV370-Quad Bead Chips	HERITAGE 20 wks; supervised; progressive, MICT; 3×/wk.; 55–75% VO_2max_; 30–50 min. DREW: 6 mths; supervised; exercise groups: 4, 8 or 12 kcal/kg/week (MICT); 3-4×/week; progressive training intensity started at 50% VO_2max_. Each group expended 4 kcal/kg/week for first week. Group 1: maintained 4 kcal/kg/week for 6 months. Group 2: increased by 1 kcal/kg/week until 8ckal/week reached – maintain for remaining time. Group 3: increased by 1 kcal/kg/week until 8ckal/week reached – maintain for remaining time. STRRIDE 1: 8–9 mths; supervised exercise sessions. Three groups: 1. High-amount/vigorous intensity exercise (170 min/week/2000 kcal/week) or the calorie equivalent of jogging for ~20 miles per week at 55–85% VO_2max_. 2. Low amount/vigorous-intensity exercise/1200 kcal/week (~120 min/week) or the equivalent of 12 miles/week for jogging at 65–80%. 3. Low amount, moderate intensity exercise (1200 kcal/week (170 min/week) or the equivalent of 12 miles/week at 40–55% VO_2max_. STRRIDE 2: 8–9 mths; supervised; four groups: 1: Aerobic training – 1300 cal – 65-80%; 2: Resistance training only with 3 sets of 12–15 reps 3 x /week. 3: Combination of the first 2 protocols; 4: High anaerobic training – 2200 cal – 3 x week – 65-80%. First 2–3 months ‘ramp up period’. Following 6 mths using appropriate protocol.
McKenzie, 2011, USA	*AKT*	Single group, longitudinal. VO_2max_ tested pre & post intervention; dietary stabilisation.	*N* = 51 M and 58 F Caucasians; 50–75 yrs.; no major health concerns; non-smoking; BMI <37; haematocrit >35; BP between 120/80 but less than 160/100 mmHg; at least one lipid abnormality; not any medication for blood pressure, cholesterol or glucose; F post-menopausal for at least 2 years (stable HRT or non HRT); inactive.	Peripheral blood leucocytes	TaqMan allelic discrimination assay using qPCR	24 wks; supervised; progressive MICT; 3×/wk.; 50–70% HRR; 20–40 min.
Thomaes, 2011, Belgium	*AMPD1; GR; CNTF*	Retrospective, single group, longitudinal. VO_2peak_ tested pre & post intervention.	N = 935 coronary artery disease patients (CAD); 76 females; Caucasian; age 56 ± 0.3 yrs.; BMI 25.8 ± 0.1 kg/m^2^; 5% smokers; 85% cardiac medications; 5% diabetes; 27% hypertension.	Peripheral blood leucocytes	Invader TM assay (third wave technologies)	3 mths; supervised; 2-3×/wk.; 80% HR_max_; 90 mins/session.
Onkelinx, 2011, Belgium	*NOS3; Catalase; VEGF; Eco-SOD; GPX; P22Phox; PPARGC1; PPARα*	Retrospective, single group, longitudinal. VO_2peak_ tested pre & post intervention.	*N* = 935 coronary artery disease patients (CAD); 76 females; Caucasian; age 56 ± 0.3 yrs.; BMI 25.8 ± 0.1 kg/m^2^; 5% smokers; 85% cardiac medications; 5% diabetes; 27% hypertension.	Peripheral blood leucocytes	Invader TM Assay (third wave technologies)	CARAGENE: 3 mths; supervised; 3×/week; 90 mins; ~ intensity = 80% (HR/peakHRx100)
Silva, 2011, Brazil	*NOS3*	Single group, longitudinal. VO_2peak_ tested pre & post intervention.	*N* = 80 Portuguese police recruits; 20–35 years; BMI 23.3 ± 3.6 kg/m^2^; no health concerns; inactive.	Peripheral blood leucocytes	PCR-RFLP	18 weeks; supervised; 3×/week/ 80 mins; intensity graded to VT HR.
Timmons, 2010, UK	*GWAS*	1: Single group, longitudinal. VO_2max_ & muscle biopsies tested pre & post intervention; 2: Blind test. VO_2max_ & muscle biopsies tested pre & post intervention; 3: Retrospective: HERITAGE WHITES data	1: N = 24 sedentary healthy Caucasian men (23 ± 1 yrs., 1.82 ± 0.02 m, 78.6 ± 2.7 kg); 2: 17 active & healthy Caucasian men (29 ± 6 yrs., 81.8 ± 9 kg, 1.8 ± 0.5 m); 3: HERITAGE Caucasians (as described in Bouchard 2011).	Lymphoblastoid cell lines from venous blood	Illumina Human CNV370-Quad Bead Chips	1: 6 weeks; supervised MICT; 4 × 45 min cycling sessions/week @ 70% VO_2max_. 2:12 weeks; cycle ergometer 5×/week. Peak power test performed every Mon to determine intensity for week: Tues: 3 min intervals at 85%. P_max_ separated by 3 min intervals at 40% P_max_; Thurs: 8 min intervals at 85% P_max_ separated by 3 min intervals at 40% P_max_; Fri: 120 min at 55% P_max_ continuously; duration increased by 5%/wk.; last 6 wks duration maintained but intensity increased by 1%/week; 3: HERITAGE WHITES Study (as described in Bouchard 2011).
Jenkins, 2010, USA	*PLIN haplotypes*	Retrospective, single group, longitudinal. VO_2max_ tested; body composition; pre & post intervention; dietary stabilisation (American Heart Association).	N = 46 M & 55 F Caucasians (50–75 years); inactive; no major health concerns; BP < 160/99; non-smokers; BMI < 37 kg/m^2^; no meds for BP, cholesterol or glucose control; at least one lipid abnormality.	Unknown	TaqMan allelic discrimination assay using qPCR	24 weeks; supervised; multi-modal MICT; progressive; 3×/wk.; 20–40 min; up to 70% VO_2max_ reached; 60 min walk home included post 12 wks.
Alves, 2009, Brazil	*ACE & Angiotensin*	Single group, longitudinal. VO_2max_ and echocardiography of left ventricle pre and post intervention.	*N* = 83 Brazilian policemen; age 26 years ±4.5; BMI 24 kg/m^2^ ± 1; healthy; normotensive.	Unknown	Polymerase chain reaction protocol.	17 weeks; supervised MICT; 50–80%VO_2peak_; 60 min × 3/week.
He, 2008a, China	*NRF-1*	Single group, longitudinal; VO_2max_, VT and RE tested pre & post intervention.	*N* = 102 Chinese male soldiers; no health concerns; age 18.8 ± 0.9 yrs.; wt 60.3 ± 6.5 kg; ht. 1.71 ± 5.8 m; no medications; non-smokers.	Peripheral blood leucocytes	PCR-RFLP assay	18 wks; supervised; 3×5000m running sessions/wk.; 95%–105% VT.
He, 2008b, China	*PPARGC1*	Single group, longitudinal; VO_2max_, VT and RE tested pre & post intervention.	N = 102 Chinese male soldiers; no health concerns; age 18.8 ± 0.9 yrs.; wt 60.3 ± 6.5 kg; ht. 1.71 ± 5.8 m; no medications; non-smokers.	Peripheral blood leucocytes	PCR-RFLP assay	18 wks; supervised; 3×5000m running sessions/wk.; 95%–105% VT.
He, 2007a, China	*TFAM*	Single group, longitudinal. VO_2max,_ VT and RE tested pre & post intervention.	N = 102 Chinese male soldiers; no health concerns; age 18.8 ± 0.9 yrs.; wt 60.3 ± 6.5 kg; ht. 1.71 ± 5.8 m; no medications; non-smokers.	Peripheral blood leucocytes	PCR-RFLP assay	18 wks; supervised; 3×5000m running sessions/wk.; 95%–105% VT.
He, 2007b, China	*NRF-2/NFE2L2*	Single group, longitudinal. VO_2max_, VT and RE tested pre & post intervention.	N = 102 Chinese male soldiers; no health concerns; age 18.8 ± 0.9 yrs.; wt 60.3 ± 6.5 kg; ht. 1.71 ± 5.8 m; no medications; non-smokers.	Peripheral blood leucocytes	PCR-RFLP assay	18 wks; supervised; 3×5000m running sessions/wk.; 95%–105% VT.
Hautala, 2007, USA	*PPARD*	Retrospective, single group, longitudinal. VO_2max_, body composition and lipids tested pre & post intervention.	*N* = 477 from HERITAGE Caucasian study (183 female) *N* = 264 from HERITAGE African-American study (247 female)	Unknown	SNP scorer genotyping software	20 wks; supervised; progressive, MICT; 3×/wk.; 55–75% VO_2max_; 30–50 min.
Defoor, 2006a, Belgium	*ADRB1*	Retrospective, single group, longitudinal. VO_2peak_ tested pre & post intervention.	N = 935 coronary artery disease patients (CAD); 76 females; Caucasian; age 56 ± 0.3 yrs.; BMI 25.8 ± 0.1 kg/m^2^; 5% smokers; 85% cardiac medications; 5% diabetes; 27% hypertension.	Peripheral blood leucocytes	Invader assay	CARAGENE: 3 mths; supervised; 2-3×/wk.; 80% HR_max_; 90 mins/session.
Defoor, 2006b, Belgium	*ACE*	Retrospective, single group, longitudinal. VO_2peak_ tested pre & post intervention.	*N* = 935 coronary artery disease patients (CAD); 76 females; Caucasian; age 56 ± 0.3 yrs.; BMI 25.8 ± 0.1 kg/m^2^; 5% smokers; 85% cardiac medications; 5% diabetes; 27% hypertension.	Peripheral blood leucocytes	Invader assay	CARAGENE: 3 mths; supervised; 2-3×/wk.; 80% HR_max_; 90 mins/session.
He, 2006, China	*HBB*	Retrospective, single group, longitudinal. VO_2max,_ VT and RE tested pre & post intervention.	N = 102 Chinese male soldiers; no health concerns; age 18.8 ± 0.9 yrs.; wt 60.3 ± 6.5 kg; ht. 1.71 ± 5.8 m; no medications; non-smokers	Peripheral blood leucocytes	PCR-RFLP assay	18 wks; supervised; 3x5000m running sessions/wk.; 95%–105% VT
Defoor, 2005	*CKMM*	Retrospective, single group, longitudinal. VO_2peak_ tested pre & post intervention.	N = 935 coronary artery disease patients (CAD); 76 females; Caucasian; age 56 yrs. ± 0.3; BMI 25.8 kg/m^2^ ± 0.1; 5% smokers; 85% cardiac medications; 5% diabetes; 27% hypertension.	Peripheral blood leucocytes	Invader assay	CARAGENE: 3 mths; supervised; 2-3×/wk.; 80% HR_max_; 90 mins/session.
Leon, 2004, USA	*APOE*	Retrospective, single group, longitudinal. VO_2max_, blood lipids tested pre & post intervention; counselled not to alter health habits.	*N* = 241 male and 89 female HERTIAGE Caucasians; 17–65 years; inactive; no major health concerns	Lymphoblastoid cell lines from venous blood	PCR-RFLP assay	HERTIAGE: 20 wks; supervised; progressive MICT; 3×/wk.; 55–75% VO_2max_; 30–50 min.
Thompson, 2004, USA	*APOE*	Single group, longitudinal. VO_2max_, anthropometric data and lipid levels collected pre & post intervention; dietary control.	*N* = 170 Caucasians (120 completed program – 60 M and F); 18–70 years (39 ± 11 years); consumed less than 2 drinks/day; physically inactive; BMI <31; no major health concerns.	Peripheral blood leucocytes	PCR-RFLP assay	6 months supervised progressive training; 60–80% of VO_2max_; increasing from 15 to 40 mins during first 4 wks. Once at 40 mins, maintained this for 4 sessions each week for 5–6 months. Multimodal but treadmill primary aerobic activity.
Rico-Sanz, 2003, Canada	*AMPD1*	Retrospective, single group, longitudinal. VO_2max_, submax and submax to maximal tested pre & post intervention.	*N* = 329 HERTAGE Caucasians and 90 HERITGAE African-Americans measured for training response; 17–65 years; inactive; no major health concerns.	Unknown	PCR protocol + separation on agarose gels	HERITAGE: 20 wks; supervised; progressive MICT; 3×/wk.; 55–75% VO_2max_; 30–50 min
Prior, 2003, USA	*HIF1A*	Single group, longitudinal. VO_2max_ tested pre & post intervention.	*N* = 101 Caucasian and 22 African-Americans in good health; age 57.7 ± 0.91 yrs.; BMI 29.2 ± 0.64 kg/m^2^	Peripheral blood lymphocytes	PCR-RFLP assay	24 weeks; supervised; progressive MICT; 3×/wk.; 20–40 min; 50–70% VO_2max_
Woods, 2002, UK	*ACE*	Single group, longitudinal. VO_2max,_ and HR/VO_2_ relationship tested pre & post intervention.	*N* = 59 Caucasians with ACE II allele and 29 without ACE DD allele; ~age 18.9 yrs.; ~ht. 1.78 m; ~ wt 73.4 kg; military camp.	Peripheral blood leucocytes	PCR protocol + polyacrylamide gel separation	11 weeks; supervised aerobic training; 75% squads; 35% adventurous training; 25% running and circuit training.
Murakami, 2001, Japan	*MtDNA*	Single group, longitudinal. VO_2max_ tested pre & post intervention	*N* = 41 Japanese M (age 20.6 ± 2.2 yrs), inactive; no major health concerns; wt 62.8 ± 7.5 kg; ht. 171.8 ± 6.7 cm.	Peripheral blood leucocytes	PCR-RFLP assay	8 weeks; supervised 1×/week out of 3.5; 60 min/session; 70% VO_2max_
Sonna, 2001, USA	*ACE*	Double-blind study. VO_2peak,_ anthropometrics physical fitness assessment for active duty personnel tested pre and post intervention.	*N* = 85 F and 62 M; age 21.7 ± 3.6 yrs.; 84 Caucasian, 20 Hispanic, 1 Native Americans, 5 Asian and 37 African-American; no major health concerns; BMI 23.1 ± 3.1 kg/m^2^; BF% 27.9 ± 6.1 F and 16.4 ± 5.7 M.	Peripheral blood leucocytes	PCR-RFLP assay	8 weeks supervised; 6 days/week; 2 x aerobic (sprints & 3–5 miles) & 2 x strength. Participants place in 1 of 4 ability groups so all running for same duration. Participants also completed road marches and other drills.
Rankinen, 2000a, USA	*Na + −K + ATPaseα*	Retrospective, single group, longitudinal. VO_2max_ and max power output tested pre & post intervention.	HERITAGE WHITES: 472 Caucasians; 17–65 years; inactive; no major health concerns.	Lympohblastoid cell lines	PCR protocol + agarose gel separation	HERTIAGE: 20 wks; supervised; progressive MICT; 3×/wk.; 55–75% VO_2max_; 30–50 min
Rankinen,2000b, USA	*ACE*	Retrospective, single group, longitudinal. V0_2max_, VE, VT, blood lactate, oxygen, stroke volume, carbon dioxide, HR, tested pre & post intervention (submax VO_2_ test for older patients).	HERITAGE WHITES AND BLACKS: 476 Caucasian & 248 Blacks; 17–65 years; inactive; no major health concerns.	Lympohblastoid cell lines	PCR protocol + agarose gel separation	HERTIAGE: 20 wks; supervised; progressive MICT; 3×/wk.; 55–75% VO_2max_; 30–50 min
Hagberg, USA, 1999	*APOE*	Retrospective, single group, longitudinal. VO_2max_ and lipid levels tested pre and post; stabilised on American Heart Association diet 8 weeks prior to intervention.	*N* = 51; 40–80-year-old sedentary men (61 ± 3 yrs); overweight with ~BF% 30 ± 3; BP < 160/95 mmHg; no major health concerns or medications for blood lipids or glucose.	Peripheral blood leucocytes	PCR-RFLP assay	9 months’ endurance training; multimodal; 5–7 months supervised and last 2–4 months used heart rate monitor to ensure 70–80% VO_2max_ intensity and 3 days/week for 45 min was complied with.
Rivera, 1999, Canada	*CKMM*	Retrospective, single group, longitudinal. VO_2max_ tested pre & post intervention.	HERITAGE WHITES: 495 Caucasians from 98 families; 17–65 years; inactive; no major health concerns.	Lympohblastoid cell lines	PCR-RFLP assay	HERTIAGE: 20 wks; supervised; progressive MICT; 3×/wk.; 55–75% VO_2max_; 30–50 min
Rivera, 1997, Canada	*CKMM*	Retrospective, single group, longitudinal. VO_2max_ tested pre & post intervention.	HERITAGE WHITES: 160 Caucasian parents and 80 offspring; 17–65 years; inactive; no major health concerns.	Lympohblastoid cell lines	PCR-RFLP assay	HERTIAGE: 20 wks; supervised; progressive MICT; 3×/wk.; 55–75% VO_2max_; 30–50 min
Dionne, 1991, Canada	*mtDNA*	Single group, longitudinal. VO_2max_ tested pre & post intervention.	N = 46 M from Quebec (17–27 yrs) & 27 M from Tempe (24–29 yrs); inactive	Peripheral blood leucocytes	PCR-RFLP assay	Quebec: 20 weeks; supervised; progressive training; Max 85% HRR; max 45 min/session; 3×/wk. Tempe: 12 weeks; supervised; progressive training; max 70–77% VO_2max_; max 40 min/session; 3×/wk
Bouchard, 1989, Canada	*AK1M* *CKM*	RCT. VO2max, total power output tested pre & post intervention.	*N* = 295 M 7 F (18–30 years); healthy Caucasians	Muscle biopsy and peripheral blood leucocytes	Formazan technique?	Group 1: 15 weeks; supervised; progressive MICT; 30–45 min/session; 3-5×/wk.; 60–85% HRR Group 2: 15 weeks; supervised; progressive interval training; 1-2×/week; 80–85% HRR separated by 5 min recovery.

*M* male, *F* female, *wks* weeks, *mths* months, *wt* weight, *ht.* height, *yrs.* years, *BMI* body mas index, *BF %* body fat percentage, *VO*_*2max*_ maximal oxygen uptake/cardiorespiratory fitness, *PCR* polymerase chain reaction protocol, *RFLP* restriction fragment length polymorphism, *qPCR* Quantatitive Polymerase Chain Reaction, *RCT* randomised controlled trial, *GWAS* genome wide association study, *HRT* hormone replacement therapy, *SNP* single nucleotide polymorphism, *AT* anaerobic threshold, *MICT* moderate intensity interval training, *HR* heart rate, *HRR* heart rate reserve, *HR*_*max*_ heart rate maximum, *P*_*max*_ maximal aerobic power, *Submax* submaximal, *Cal/kcal* calories, *mtDNA* mitochondrial DNA, *BP* blood pressure

A summary of key findings from the included articles is provided in Tables 2 and 3. Limitations were assessed by two authors (CW and JC) based on the intervention, genotyping method used, study design and sample used. Table 4 was developed to highlight which predictor genes for VO_2max_ trainability merited further exploration. A third author (MW) examined Tables 1, 2, 3 and 4 to ensure all genetic variants, genomic coordinates and genotypes, were described with a consistent annotation.

**Table 2:** Summary of findings from candidate gene studies

Gene	Variant	Chromosome	Author & Date	Race	Age	Sex	Health concerns	(+/−/0)* Genotype & VO_2max_ training response	P-value (x)	Highest training intensity	Sessions/week	Duration per session (min)	Training period	Training modality
*PPARGC1*	Intron 7G/C	22	Onkelinx, 2011	935 Caucasian	~56	M&F	Y (CAD)	GG, CG, CC (0)	0.51	80% HRmax	2–3	90	3 months	Ambulatory
			He, 2008b	102 Chinese	~19	M	N	All variants (0)	> 0.05	95–105% VT	3	Time to finish.	18 weeks	5000 m running
*APOE*	E2: rs7412 (c.526C > T; p.Arg176Cys) E3: WT E4: rs429358 (c.388 T > C; p.Cys130Arg) E3/E3: WT/WT E2/E3: p.Arg176Cys/WT E4/E3: p.Cys130Arg/WT E2/E2: p.Arg176Cys/p.Arg176Cys E2/E4: p.Arg176Cys/p.Cys130Arg E4/E4: p.Cys130Arg/p.Cys130Arg	19	Yu, 2014	360 Chinese	18–40	M F M F M&F	N	E2/E3 in M (+) n = 20 E2/E3 F (+) n = 25 E3/E4 M (+) n = 31 E3/E4 F (+) n = 29 E2/E2; E2/E4; E3/E3; E4/E4 in M&F (0)	0.04 0.03 0.02 0.02 > 0.05	60–85% VO_2max_	‘Progressive’ but details NA	‘Progressive’ but details NA	6 months	Treadmill
			Leon, 2004	265 Caucasian	17–65	M&F	N	All variants (0)	> 0.05	75% VO_2max_	3	30–50	20 weeks	Cycle ergo
			Thompson, 2004	170 Unknown	~39	M&F	N	E3/E3 (−) n = 43 E2/E3 (0) n = 40 E3/E4 (0) n = 41	< 0.01	60–85% VO_2max_	4	Up to 50 min	6 months	Treadmill
*CKM*	1170 & 985 + 185	19	Defoor, 2005	935 Caucasian	~56	M&F	Y (CAD)	AA; GG; A/G (0)	> 0.05	80% HRmax	2–3	90	3 months	Ambulatory
			Rivera, 1999	240 Caucasian	17–65	M&F	N	CKM locus (*n* = 227)	< 0.01	75% VO_2max_	3	30–50	20 weeks	Cycle ergo
			Rivera, 1997	495 Caucasian	17–65	M&F	N	Homozygotes 1170bpa allele (−) *n* = 12	< 0.05	75% VO_2max_	3	30–50	20 weeks	Cycle ergo
			Bouchard, 1989	295 Caucasian	18–30	M&F	N	All variants (0)	> 0.05	1. 60–85% HRR 2: 80–85% HRR	1: 1–2 2: 3–5	1: Intervals 2: 30–45	1: 15 2: 15	1: Cycling 2: Cycling
*ACE*	Insertion (I) or Deletion (D)	17	Alves, 2009	83 Brazilian	~26	M	N	All variants (0)	> 0.05	50–80% VO_2peak_	2–3	60 min	17 weeks	Running
			Rankinen, 2000b	476 Caucasian 248 AA	17–65	M&F	N	DD Caucasian offspring (+) *n* = 81	0.042	75% VO_2max_	3	30–50	20 weeks	Ergo cycle
			Defoor, 2006	935 Caucasian	~56	M&F	Y (CAD)	II (+) (frequency of 0.3 M and 0.36 F)	Entire group: 0.047 No Ace inhibitors: 0.013	80% HRmax	2–3	90	3 months	Ambulatory
			Woods, 2002	59 Caucasian	~19	M	N	II; I/D; DD (0)	>0.22	NA	NA	NA	11 weeks	Squads, adventure training, running, circuits
			Sonna, 2001	147 Caucasian, 37 AA, 26 other	19–24	M&F	N	II, DD (0)	>0.05	NA	4–6	90 min	8 weeks	Military training
*CYBA; P22Phox*	A24G – 640A > G	16	Onkelinx, 2011	935 Caucasian	~56	M&F	Y (CAD)	AA, AG, GG (0) CC, CT, TT (0)	0.78 0.94	80% HRmax	2–3	90	3 months	Ambulatory
*PLIN*	PLIN1 (6209 T > C) – rs2289487 15:g.90217096C > T PLIN4 (11482G > A) – rs894160 15:g.90211823C > T PLIN5 (13041A > G – rs2304795 15:g.90210263A > G PLIN6 (149954A > T – rs1052700 15:g.90208310A > T	15	Jenkins, 2010	101 Caucasian	NA	M&F	N	Genotypes and haplotypes (0)	*p* > 0.05	Up to 70% VO_2max_	3	20–40 min	24 weeks	Multi-modal
*AKT*	rs1130214 (4:g.105259734C > A)	14	McKenzie, 2011	109 Caucasian	50–75	M F	Elevated BP, cholesterol, menopause	All genotypes sig. Increased, but GT/TT men (+) *n* = 22	0.037	50–70%HRR	3	20–40 min	24 weeks	Multi-modal
*HIF1A*	T + 140C (rs11549465) A-2500 T	Ch 14	Prior, 2003	101 Caucasian 22 AA	>60 <60	M&F	N	CT & TT in Caucasian over 60 (−) *n* = 37 All other ages, race and genotypes (0)	0.03 >0.05 >0.05	50–70% VO_2max_	3	20–40 min	24 weeks	‘Aerobic training’
*Na + −K + −ATPase α2*	Alpha2 exon 1 Alpha2 exon 21–22	13	Rankinen, 2000a	472 Caucasian	17–65	M&F	N	3.3/3.3 (−) *n* = 5 10.5/10.5 offspring (+) *n* = 14	0.018 0.017	55–75% VO_2max_	3	30–50	20 weeks	Cycle ergo
*HBB*	-551C/T – no rs ID 11:g.5248801 T > C +16, intron 2 - rs10768683 11:g.5247791C > G +340 – no rs ID 11:g.5246488 T > A	11	He, 2006	102 Chinese	~19	M	N	CC, CT, TT (0) CC, CG, GG (0) AA, AT, TT (0)	>0.05	95–105% VT	3	Time to finish.	18 weeks	5000 m running
*CNTF*	rs1800169 (11:g.58391501G > A)	11	Thomaes, 2011	935 Caucasian	~56	M&F	N	AA (+) *n* = 21	0.002	80% HR max	2–3	90	3 months	Ambulatory
*CAT*	-262C > T	11	Onkelinx, 2011	935 Caucasian	~56	M&F	Y (CAD)	TT (−) *n* = 342	0.02	80% HR max	2–3	90	3 months	Ambulatory
*GSTP1*	rs1695 (11:g67352689A > G c.313A > G p.Ile105Val)	11	Zabreska, 2014	66 Polish	19–24	F	N	GG & AG (+) *n* = 30	Absolute: 0.029 Relative: 0.025	50–75% HR max	3	60	3 months	‘Aerobic routine’
*ADRB1*	Pos. 145 Pos. 1165	10	Defoor, 2006	935 Caucasian	~56	M&F	Y(CAD)	Ser49Gly49, Ser49Ser49,		80% HR max	2–3	90	3 months	Ambulatory
								Gly49Gly49 (0) GLy389Gly389,	0.18					
								Gly389Arg389, Arg389Arg389 (0)	0.75					
*TFAM*	rs1937 (10:g.60145342G > C c.35G > C p.Ser12Thr) rs2306604 (10:g.60148692A > G) rs1049432 (10:g.60155120G > T)	10	He, 2007b	102 Chinese	~19	M	N	GG, CG, CC (0) AA, AG, GG (0) GG, GT, TT (0)	>0.05	95–105% VT	3	Time to finish.	18 weeks	5000 m running
*NOS3*	T-1495A – No rs ID 7:g.150689397A > T A-949G – rs1800779 7:g.150689943G > A -786 T > C– rs41322052 7:g150690106C > T G298A – rs1799983 7:g.150696111 T > G c.894 T > G (p.Asp298Glu))	7	Onkelinx, 2011	935 Caucasian	~56	M&F	Y (CAD)	TT, TA, AA (0) AA, AG, GG (0) TT, TC, CC (0) TT, CT, C (0) CC, CT, TT (0) GG, GA, AA (0)	0.54 0.76 0.69 0.69 1.88 1.04	80% HRmax	2–3	90	3 months	Ambulatory
	-786 T > C– rs41322052 7:g150690106C > T Intron 4 – rs61722009 VNTR (repeat) 7:g.150694276_150694302AGGGGTG 894G > T – rs1799983 7:g.150696111 T > G c.894 T > G (p.Asp298Glu))	7	Silva, 2011	80 Portuguese	20–35	M	N	TT, CC, TC (0) 4b4b, 4ba4c, 4a4a (0) GG, GT, TT (0) *All genotypes sig. Increased. fitness, thus no difference between groups	0.001	Graded to VT HR	3	80 min	18 weeks	Running
*NRF-1*	C&T - rs2402970 7:g.80647382G > T A & G - rs10500120 7:g.129393341A > G rs6949152 7:g129286436A > G	7	He, 2008a	102 Chinese	~19	M	N	CC, CT, TT (0) AA, AG, GG (0) AA, AG, GG (0)	0.38 0.110 0.094	95–105% VT	3	Time to finish.	18 weeks	5000 m running
*AK1M*	common and rare variants	7	Bouchard, 1989	295 Caucasian	18–30	M&F	N	(0)	> 0.05	1. 85% HRR 2: 85% HRR	1: 1–2 2: 3–5	1: Intervals 2: 30–45	1: 15 2: 15	1: Cycling 2: Cycling
*PPARD*	Exon 4 + 15 Exon 7 + 65	Ch 6	Hautala, 2007	Caucasian AA	17–65	M&F	N	CC genotype in AA of Exon 4 + 15 (−) *n* = 19	0.005	75% VO_2max_	3	30–50	20 weeks	Cycle ergo
*VEGF*	405 460	6	Onkelinx, 2011	935 Caucasian	~56	M&F	Y (CAD)	GG, GC, CC (0) CC, CT, TT (0)	0.52 0.52	80% HR max	2–3	90	3 months	Ambulatory
*GR/NR3C1*	rs6190 (5:g.142780337C > T c.68G > A p.Arg23Lys)	5	Thomaes, 2011	935 Caucasian	~56	M&F	Y (CAD)	G/A (+) *n* = 55	<0.01	80% HR max	2–3	90	3 months	Ambulatory
*PPARα*	Gly482Ser	4	Onkelinx, 2011	935 Caucasian	~56	M&F	Y (CAD)	GG, G, SS (0)	0.59 0.8	80% HR max	2–3	90	3 months	Ambulatory
*SOD3*	C760G	4	Onkelinx, 2011	935 Caucasian	~56	M&F	Y (CAD)	CC (0) G carrier (0)	0.12 0.18	80% HR max	2–3	90	3 months	Ambulatory
*GPX*	197P > L	3	Onkelinx, 2011	935 Caucasian	~56	M&F	Y(CAD)	Pro197Pro (0) Leu-carrier (0)	0.18 0.78	80% HR max	2–3	90	3 months	Ambulatory
*NFE2L2*	Rs125949 Rs8031031 Rs718186	2	He, 2007b	102 Chinese	~19	M	N	CC, CA, AA (0) CT, TT, AA (0) AG, GG (0)	> 0.05	95–105% VT	3	Time to finish.	18 weeks	5000 m running
*AMPD1*	AMPD1:c.133C (rs17602729)	1	Thomaes, 2011	935 Caucasian	~56	M&F	N	CC (+) *n* = 652	< 0.05	80% HR max	2–3	90	3 months	Ambulatory
			Rico-Sanz, 2003	329 Caucasian 90 AA	17–65	M&F	N	TT (−) in Caucasians (*n* = 6)	< 0.006	75% VO_2max_	3	30–50	20 weeks	Cycling
*mtDNA*	MTND5 m.13470A > C or A > G m.12406G > A m.13365C > T	mtDNA SNP via restriction enzyme	Murakami, 2001	21 Japanese	20.6	M	N	All variants (0)	> 0.05	70% VO_2max_	3–4	60 min	8 weeks	Ergo Cycle
*mtDNA*		Within mitochondria	Dionne, 1991	53 Quebec, Tempe	17–27	M	N	mtDNA subunit 5 N5 (−) *n* = 3	0.05	Quebec: 85% HRR Tempe:77% VO_2max_	Quebec: 3 Tempe: 3–5	Quebec:45 min Tempe:40 min	Quebec: 20 wks Tempe: 12 wks	Ergo Cycle
*ALAS2*	≤166 bp	Mitochondria	Xu, 2015	72 Chinese	18–22	M	N	≤166 bp (+) *n* = 25	< 0.05	‘High/Low training’	3	30 min	4 weeks	Ergo Cycle

where possible, gene variants were annotated using the references sequence (GRCh37/hg19)

*CAD* coronary artery disease, *wks* weeks, *mths* months, *VO*_*2max*_ maximal oxygen uptake/cardiorespiratory fitness, *AT* anaerobic threshold, *HRR* heart rate reserve, *HRmax* heart rate maximum, *Pmax* maximal aerobic power, *Cauc* Caucasian, *AA* African-American, *M* male, *F* female

**(+) = high training response, (−) = low training response, (0) = neutral training response

(x) = p-value has been adjusted for covariates except for article by Xu et al. (2015) where it wasn’t clear if p-value had been adjusted (ALAS_2_)

**Table 3:** Summary of hypothesis-free studies

Gene	Variant	Chromosome	MapPosition	Minor allele frequency (MAF) frequency	Race	Gender	Age	Training period	Sessions/wk	Session duration	Sessions intensity	(+/−/0)** genotype/expression and VO2max response to training	P-value	Author, Date
^*CAMTA1 intronic	*rs884736*	*1*	*6,937,692*	*0.41*	*1. 473 Caucasian* *2. 259 African-American*	*M&F* *M&F*	*17–65* *17–65*	*20 wks*	*3×/wk*	*30–50 min*	*55–75% VO*_*2*_ *max*	*AA (−)*	*1. 1.49* × *10-* ^4^ *2. 0.03* *3. 1.54 × 10* ^*−4*^	*Bouchard, 2011 (1&2)* *Ghosh, 2013 (3)*
+ID3	rs11574 (1:g.23559007 T > C c.313A > G p. Thr105Ala)	1	23,758,085	NA	473 Caucasian	M&F	17–65	20 wks	3×/wk	30–50 min	55–75% VO_2_max	NA	2.1 × 10^−3^	Timmons, 2010
*RGS18 *5′ upstream of gene (non-coding)*	*rs10921078 (1:g.192059022G > A)*	*1*	*190,325,645*	*0.15*	*1. 483 Caucasian* *2. 259 African-American*	*M&F*	*17–65*	*20 wks*	*3×/wk*	*30–50 min*	*55–75% VO*_*2*_ *max*	*GG (−) n = 567*	*1. 7.17* × *10~* ^5^ *2. 0.032*	*Bouchard, 2011*
**^RYR2** **intronic**	**rs7531957 (1:g.237789656 T > G)**	**1**	**235,856,279**	**0.08**	**473 Caucasian)**	**M&F**	**17–65**	**20 wks**	**3×/wk**	**30–50 min**	**55–75% VO**_**2**_ **max**	**NA**	**1:6.42** × **10–** ^**5**^ **2:1.18 × 10** ^**−4**^	**Bouchard, 2011 (1)** **Ghosh, 2013 (2)**
**#SCLC45A1**	**NA**	**1**	**NA**	**NA**	**473 Caucasian**	**M&F**	**17–65**	**20 wks**	**3×/wk**	**30–50 min**	**55–75% VO**_**2**_ **max**	**NA**	**#89.1**	**Ghosh, 2013**
MAST2	rs2236560	1	46,268,021	NA	41 Caucasian	M	Young adults	1.6 wks 2. 12 wks	1. 4×/wk. 2. 3×/wk	1. 45 min 2. Progressive	1. 70% VO_2_max 2. Progressive	NA	NA	Timmons, 2010
SYPL2	rs12049330	1	109,832,711	NA	41 Caucasian	M	Young adults	1.6 wks 2.12 wks	1. 4×/wk. 2. 3×/wk	1. 45 min 2. Progressive	1. 70% VO_2_max 2. Progressive	NA	NA	Timmons, 2010
#ACVR1C	NA	2	NA	NA	473 Caucasian	M&F	17–65	20 wks	3×/wk	30–50 min	55–75% VO_2_max	NA	#85.8	Ghosh, 2013
SLC4A5	rs828902	2	74,323,642	NA	473 Caucasian	M&F	17–65	20 wks	3×/wk	30–50 min	55–75% VO_2_max	NA	NA	Timmons, 2010
KCNF1/NLGN1	rs2003298 (2:g.11086150 T > C)	2	11,003,601	0.42	473 Caucasian	M&F	17–65	20 wks	3×/wk	30–50 min	55–75% VO_2_max	NA	1.21 × 10~^4^	Bouchard, 2011
* FLJ44450	rs4952535 (2:g.42131523G > A)	2	41,985,027	0.41	473 Caucasian	M&F	17–65	20 wks	3×/wk	30–50 min	55–75% VO_2_max	G (+)	1.01 × 10-^4^	Bouchard, 2011
++TTN	rs10497520 (2:g.179644855 T > C c3601A > G p.Lys1201Glu)	2	175,353,100	0.50	473 Caucasian	M&F	17–65	20 wks	3×/wk	30–50 min	55–75% VO_2_max	NA	2.5 × 10^−3^	Timmons, 2010
++NRP2 intronic	rs3770991 (2:g.206655739A > G)	2	206,363,984	NA	473 Caucasian	M&F	17–65	20 wks	3×/wk	30–50 min	55–75% VO_2_max	NA	1.4 × 10^−3^	Timmons, 2010
CREB1	rs2709356	2	208,120,337	NA	473 Caucasian	M&F	17–65	20 wks	3×/wk	30–50 min	55–75% VO_2_max	NA	NA	Timmons, 2010
SCN3A	rs7574918	2	165,647,425	NA	473 Caucasian	M	Young adults	1.6 wks 2. 12 wks	1. 4×/wk. 2. 3×/wk	1. 45 min 2. Progressive	1. 70% VO_2_max 2. Progressive	NA	NA	Timmons, 2010
^HCG22	rs2517512 (6:g.31029685C > T)	6	NA	0.18	473 Caucasian	M&F	17–65	20 wks	3×/wk	30–50 min	55–75% VO_2_max	NA	3.09 × 10^−5^	Ghosh, 2013
*KCNH8 (268 kb)	rs4973706 (3:g.18921772 T > C)	3	18,896,776	0.24	473 Caucasian	M&F	17–65	20 wks	3×/wk	30–50 min	55–75% VO_2_max	A (+)	5.31 × 10~^5^	Bouchard, 2011
*ZIC4 (146 kb) intronic	*rs11715829*	*3*	*148,439,856*	*0.08*	*1. 473 Caucasian* *2. 183 Caucasian*	*M&F* *M&F*	*17–65* *40–65*	*20 wks* *6 mths*	*3×/wk.* *3-4×/wk*	*30–50 min* *4-8 kcal/kg/week*	*55–75% VO*_*2*_ *max* *+50%VO*_*2*_ *max*	*AA (−) n = 4*	*8.68* × *10-* ^6^ *0.032*	*Bouchard, 2011*
*NLGN1 (110 kb) intronic	rs2030398 (3:g.173005973G > A)	3	174,488,667	0.20	473 Caucasian	M&F	17–65	20 wks	3×/wk	30–50 min	55–75% VO_2_max	A (+)	1.32 × 10~^4^	Bouchard, 2011
^ADCY	NA	3	NA	NA	473 Caucasian	M&F	17–65	20 wks	3×/wk	30–50 min	55–75% VO_2_max	NA	#86.1	Ghosh, 2013
AMOTL2	rs13322269	3	135,569,834	NA	41 Caucasian	M	Young adults	1.6 wks 2.12 wks	1. 4×/wk. 2. 3×/wk	1. 45 min 2. Progressive	1. 70% VO_2_max 2. Progressive	NA	NA	Timmons, 2010
CSN1S2B intronic	rs2272040 (4:g71007047A > G)	4	71,041,636	0.13	473 Caucasian	M&F	17–65	20 wks	3×/wk	30–50 min	55–75% VO_2_max	NA	5.05 × 10-^5^	Bouchard, 2011
*LOC100289626 (134 kb)	rs2053896 (4:g137154796G > A)	4	137,374,246	0.10	473 Caucasian	M&F	17–65	20 wks	3×/wk	30–50 min	55–75% VO_2_max	A (+)	6.62 × 10~^5^	Bouchard, 2011
^*ACSL1	**rs6552828 (4:g.185725416A > G)**	**4**	**185,962,410**	**0.37**	**473 Caucasian**	**M&F**	**17–65**	**20 wks**	**3×/wk**	**30–50 min**	**55–75% VO**_**2**_ **max**	**AA (−)**	**1:1.31** × **10–** ^**6**^ **2:3.8 × 10** ^**−6**^	**Bouchard, 2011 (1)** **Ghosh, 2013 (2)**
^SLED1	rs6552828	4	NA	NA	473 Caucasian	M&F	17–65	20 wks	3×/wk	30–50 min	55–75% VO_2_max	NA	3.8 × 10^−6^	Ghosh, 2013
^C4orf40	rs3775758 (4:g.71008910C > T)	4	NA	NA	473 Caucasian	M&F	17–65	20 wks	3×/wk	30–50 min	55–75% VO_2_max	NA	1.09 × 10^−4^	Ghosh, 2013
^TEC	rs13117386 (4:g.48252763G > C)	4	NA	NA	473 Caucasian	M&F	17–65	20 wks	3×/wk	30–50 min	55–75% VO_2_max	NA	7.97 × 10^−5^	Ghosh, 2013
#NLN	NA	5	NA	NA	473 Caucasian	M&F	17–65	20 wks	3×/wk	30–50 min	55–75% VO_2_max	NA	#88	Ghosh, 2013
FAABP6	rs7734683	5	NA	NA	473 Caucasian	M&F	17–65	20 wks	3×/wk	30–50 min	55–75% VO_2_max	NA	1.44 × 10^−4^	Ghosh, 2013
TTC1	rs2176830	5	159,380,714	0.13	473 Caucasian	M&F	17–65	20 wks	3×/wk	30–50 min	55–75% VO_2_max	NA	1.42 × 10~^4^	Bouchard, 2011
BTNL9	rs888949	5	180,425,011	NA	41 Caucasian	M	Young adults	1.6 wks 2.12 wks	1. 4×/wk. 2. 3×/wk	1. 45 min 2. Progressive	1. 70% VO_2_max 2. Progressive	NA	NA	Timmons, 2010
RTN4IP1/QRSL1	rs898896	6	107,169,855	NA	41 Caucasian	M	Young adults	1.6 wks 2.12 wks	1. 4×/wk. 2. 3×/wk	1. 45 min 2. Progressive	1. 70% VO_2_max 2. Progressive	NA	NA	Timmons, 2010
HCG22	rs2523849	6	31,133,030	0.17	473 Caucasian	M&F	17–65	20 wks	3×/wk	30–50 min	55–75% VO_2_max	NA	7.53 × 10-^5^	Bouchard, 2011
HCG22	rs2523848	6	31,133,083	0.17	473 Caucasian	M & F	17–65	20 wks	3×/wk	30–50 min	55–75% VO_2_max	NA	7.53 × 10~^5^	Bouchard, 2011
HCG22	rs2428514	6	31,135,495	0.15	473 Caucasian	M&F	17–65	20 wks	3×/wk	30–50 min	55–75% VO_2_max	NA	8.22 × 10-^5^	Bouchard, 2011
HCG22	rs2517518	6	31,136,324	0.17	473 Caucasian	M&F	17–65	20 wks	3×/wk	30–50 min	55–75% VO_2_max	NA	7.53 × 10~^5^	Bouchard, 2011
HCG22	rs2523840	6	31,138,404	0.17	473 Caucasian	M&F	17–65	20 wks	3×/wk	30–50 min	55–75% VO_2_max	NA	7.53 × 10-^5^	Bouchard, 2011
HCG22	rs2517506	6	31,139,659	0.17	473 Caucasian	M&F	17–65	20 wks	3×/wk	30–50 min	55–75% VO_2_max	NA	7.53 × 10~^5^	Bouchard, 2011
*PRDM1 (287 kb)	rs10499043	6	106,353,830	0.13	473 Caucasian	M&F	17–65	20 wks	3×/wk	30–50 min	55–75% VO_2_max	A (+)	3.93 × 10-^6^	Bouchard, 2011
*ENPP3 (17 kb)	rs10452621	6	132,127,094	0.12	473 Caucasian	M&F	17–65	20 wks	3×/wk	30–50 min	55–75% VO_2_max	A (+)	1.23 × 10~^4^	Bouchard, 2011
+SLC22A3	rs2457571	6	160,754,818	NA	473 Caucasian	M&F	17–65	20 wks	3×/wk	30–50 min	55–75% VO_2_max	Downregulated in high responders	3.0 × 10^−3^	Timmons, 2010
^TMEM181	NA	6	NA	NA	473 Caucasian	M&F	17–65	20 wks	3×/wk	30–50 min	55–75% VO_2_max	NA	#84.5	Ghosh, 2013
^PARK2	NA	6	NA	NA	473 Caucasian	M&F	17–65	20 wks	3×/wk	30–50 min	55–75% VO_2_max	NA	#84.8	Ghosh, 2013
^SNX14	NA	6	NA	NA	473 Caucasian	M&F	17–65	20 wks	3×/wk	30–50 min	55–75% VO_2_max	NA	#86.7	Ghosh, 2013
^BTBD9	NA	6	NA	NA	473 Caucasian	M&F	17–65	20 wks	3×/wk	30–50 min	55–75% VO_2_max	NA	#86	Ghosh, 2013
^KCNQ5	NA	6	NA	NA	473 Caucasian	1.M&F 2. M	1.17–65 2. young adults	1.20 wks 2. 6–12 wks	1. 3×/wk. 2. 3–4/wk	1. 30–50 min 2. 45 min vs progressive	1. 55–75% VO_2_max 2. 70% vs progressive	NA NA	1:#85.9 2:NA	Ghosh, 2013 (1), Timmons, 2010 (2)
PPARD	rs2076167	6	35,499,765	NA	473 Caucasian	M&F	17–65	20 wks	3×/wk	30–50 min	55–75% VO_2_max	NA	NA	Timmons, 2010
HDAC9	rs3814991	7	18,601,428	0.11	473 Caucasian	M&F	17–65	20 wks	3×/wk	30–50 min	55–75% VO_2_max	NA	1.46 × 10-^4^	Bouchard, 2011
WBSCR17 (35 kb)	rs12538806	7	70,200,777	0.30	473 Caucasian	M & F	17–65	20 wks	3×/wk	30–50 min	55–75% VO_2_max	NA	1.47 × 10~^4^	Bouchard, 2011
WBSCR17 (33 kb)	rs13235325	7	70,202,943	0.30	473 Caucasian	M&F	17–65	20 wks	3×/wk	30–50 min	55–75% VO_2_max	NA	1.47 × 10-^4^	Bouchard, 2011
++CPVL	rs4257918	7	29,020,374	NA	473 Caucasian	M&F	17–65	20 wks	3×/wk	30–50 min	55–75% VO_2_max	Upregulated in high responders	3.1 × 10^−3^	Timmons, 2010
^ITGB8	rs10265149	7	NA	NA	473 Caucasian	M&F	17–65	20 wks	3×/wk	30–50 min	55–75% VO_2_max	NA	7.04 × 10^−5^	Timmons, 2010
LHFPL3	NA	7	NA	NA	473 Caucasian	M&F	17–65	20 wks	3×/wk	30–50 min	55–75% VO_2_max	NA	84.34	Ghosh, 2013
PILRB	rs13228694	7	99,778,243	NA	41 Caucasian	Young adults	17–65	1.6 wks 2. 12 wks	1. 4×/wk. 2. 3×/wk	1. 45 min 2. Progressive	1. 70% VO_2_max 2. Progressive	NA	NA	Timmons, 2010
+DEPDC6	rs7386139	8	121,096,600	NA	473 Caucasian	M&F	17–65	20 wks	3×/wk	30–50 min	55–75% VO_2_max	NA	1.85×10^−2^	Timmons, 2010
**#PINX1**	**N/A**	**8**	**NA**	**NA**	**473 Caucasian**	**M&F**	**17–65**	**20 wks**	**3×/wk**	**30–50 min**	**55–75% VO**_**2**_ **max**	**NA**	**88.2**	**Ghosh, 2013**
*GRIN3A (516 kb)	rs1535628	9	104,056,570	0.09	473 Caucasian	M & F	17–65	20 wks	3×/wk	30–50 min	55–75% VO_2_max	NA	6.81 × 10~^6^	Bouchard, 2011
GRIN3A (540 kb)	rs959066	9	104,081,084	0.27	473 Caucasian	M&F	17–65	20 wks	3×/wk	30–50 min	55–75% VO_2_max	NA	1.35 × 10-^4^	Bouchard, 2011
*C9orf27 (33 kb)	rs12115454	9	117,759,871	0.11	473 Caucasian	M&F	17–65	20 wks	3×/wk	30–50 min	55–75% VO_2_max	G (+)	7.74 × 10~^5^	Bouchard, 2011
^TTLL11	rs7022103	9	NA	NA	473 Caucasian	M&F	17–65	20 wks	3×/wk	30–50 min	55–75% VO_2_max	NA	1.08 × 10^−4^	Ghosh, 2013
KCNT1	N/A	9	NA	NA	473 Caucasian	M&F	17–65	20 wks	3×/wk	30–50 min	55–75% VO_2_max	NA	#86.5	Ghosh, 2013
KLF4	rs4631527	9	109,309,857	NA	41 Caucasian	M	Young adults	1.6 wks 2. 12 wks	1. 4×/wk. 2. 3×/wk	1. 45 min 2. Progressive	1. 70% VO_2_max 2. Progressive	NA	NA	Timmons, 2010
TET1	rs12413410	10	70,055,236	NA	41 Caucasian	M	Young adults	1.6 wks 2. 12 wks	1. 4×/wk. 2. 3×/wk	1. 45 min 2. Progressive	1. 70% VO_2_max 2. Progressive	NA	NA	Timmons, 2010
PRKG1	N/A	10	NA	NA	473 Caucasian	M&F	17–65	20 wks	3×/wk	30–50 min	55–75% VO_2_max	NA	#87.3	Ghosh, 2013
^+SVIL	rs6481619	10	30,022,960	NA	473 Caucasian	M&F	17–65	20 wks	3×/wk	30–50 min	55–75% VO_2_max	NA	1.0 × 10^−3^	Timmons, 2010
+BTAF1	rs2792022	10	93.730,409	NA	473 Caucasian	M&F	17–65	20 wks	3×/wk	30–50 min	55–75% VO_2_max	NA	1.2 × 10^−2^	Timmons, 2010
CASC2	rs1413184	10	NA	NA	473 Caucasian	M&F	17–65	20 wks	3×/wk	30–50 min	55–75% VO_2_max	NA	1.65 × 10^−4^	Ghosh, 2013
KIF5B	rs806819	10	32,403,990	NA	473 Caucasian	M&F	17–65	20 wks	3×/wk	30–50 min	55–75% VO_2_max	NA	NA	Timmons, 2010
+H19	rs22551375	11	1,976,072	NA	473 Caucasian	M&F	17–65	20 wks	3×/wk	30–50 min	55–75% VO_2_max	Upregulated in high responders	4.0 × 10^−4^	Timmons, 2010
ACTN3	rs1815739	10	66,084,671	NA	473 Caucasian	M&F	17–65	20 wks	3×/wk	30–50 min	55–75% VO_2_max	NA	NA	Timmons, 2010
BTAF1	rs2792022	10	93,730,409	NA	41 Caucasian	M	Young adults	1.6 wks 2. 12 wks	1. 4×/wk. 2. 3×/wk	1. 45 min 2. Progressive	1. 70% VO_2_max 2. Progressive	NA	NA	Timmons, 2010
*LOC100130460	rs2198009	11	10,360,153	0.50	473 Caucasian	M&F	17–65	20 wks	3×/wk	30–50 min	55–75% VO_2_max	A (+)	2.28 × 10-^5^	Bouchard, 2011
*DBX1 (64 kb)	rs10500872	11	20,202,299	0.15	473 Caucasian	M & F	17–65	20 wks	3×/wk	30–50 min	55–75% VO_2_max	A (+)	6.49 × 10~^6^	Bouchard, 2011
^*CD44	**rs353625**	**11**	**35,125,122**	**0.32**	**473 Caucasian**	**M&F**	**17–65**	**20 wks**	**3×/wk**	**30–50 min**	**55–75% VO**_**2**_ **max**	**NA**	**1:1.12** × **10–** ^**4**^ **2:1.64 × 10** ^**−4**^	**Bouchard, 2011 (1)** **Ghosh, 2013 (2)**
CXCR5 (36 kb)	rs4938561	11	118,223,695	0.23	473 Caucasian	M&F	17–65	20 wks	3×/wk	30–50 min	55–75% VO_2_max	NA	9.29 × 10~^5^	Bouchard, 2011
* CXCR5 (24 kb/) BLR1	rs7933007	11	118,235,879	0.23	473 Caucasian	M&F	17–65	20 wks	3×/wk	30–50 min	55–75% VO_2_max	NA	7.35 × 10-^5^	Bouchard, 2011
^CD6	rs175098	11	NA	NA	473 Caucasian	M&F	17–65	20 wks	3×/wk	30–50 min	55–75% VO_2_max	NA	1.11 × 10^−4^	Ghosh, 2013
**^SHANK2**	**rs10751308**	**11**	**NA**	**NA**	**473 Caucasian**	**M&F**	**17–65**	**20 wks**	**3×/wk**	**30–50 min**	**55–75% VO**_**2**_ **max**	**NA**	**8.11 × 10** ^**−5**^	**Ghosh, 2013**
**#GRIK4**	**N/A**	**11**	**NA**	**NA**	**473 Caucasian**	**M&F**	**17–65**	**20 wks**	**3×/wk**	**30–50 min**	**55–75% VO**_**2**_ **max**	**NA**	**88.32**	**Ghosh, 2013**
H19	rs2251375	11	1,976,076	NA	41 Caucasian	M	Young adults	1.6 wks 2. 12 wks	1. 4×/wk. 2. 3×/wk	1. 45 min 2. Progressive	1. 70% VO_2_max 2. Progressive	NA	NA	Timmons, 2010
FAM19A2	rs2168452	12	NA	NA	473 Caucasian	M&F	17–65	20 wks	3×/wk	30–50 min	55–75% VO_2_max	NA	1.34 × 10^−4^	Ghosh, 2013
^C12orf36 (14 kb)	rs12580476	12	13,435,330	0.14	473 Caucasian	M&F	17–65	20 wks	3×/wk	30–50 min	55–75% VO_2_max	NA	1.08 × 10~^4^ 2. 1.45 × 10^−4^	Bouchard, 2011 (1) Ghosh, 2013 (2)
**^NALCN**	**N/A**	**13**	**NA**	**NA**	**473 Caucasian**	**M&F**	**17–65**	**20 wks**	**3×/wk**	**30–50 min**	**55–75% VO**_**2**_ **max**	**NA**	**#85**	**Ghosh, 2013**
+MIPEP	rs7324557	13	23,194,862	NA	473 Caucasian	M&F	17–65	20 wks	3×/wk	30–50 min	55–75% VO_2_max	NA	5.1 × 10^−3^	Timmons, 2010
^EEF1DP3	rs2773968	13	NA	NA	473 Caucasian	M&F	17–65	20 wks	3×/wk	30–50 min	55–75% VO_2_max	NA	3.67 × 10^−6^	Ghosh, 2013
^CLYBL	N/A	13	NA	NA	473 Caucasian	M&F	17–65	20 wks	3×/wk	30–50 min	55–75% VO_2_max	NA	#85.4	Ghosh, 2013
*TTC6	rs12896790	14	37,343,673	0.09	473 Caucasian	M&F	17–65	20 wks	3×/wk	30–50 min	55–75% VO_2_max	NA	3.59 × 10-^5^	Bouchard, 2011
METTL3	rs1263809	14	21,058,740	NA	41 Caucasian	M	Young adults	1.6 wks 2. 12 wks	1. 4×/wk. 2. 3×/wk	1. 45 min 2. Progressive	1. 70% VO_2_max 2. Progressive	NA	NA	Timmons, 2010
TTC6	rs8018889	14	37,353,342	0.09	473 Caucasian	M & F	17–65	20 wks	3×/wk	30–50 min	55–75% VO_2_max	NA	5.25 × 10~^5^	Bouchard, 2011
*DAAM1	*rs1956197 (14:g.59477414C > T)*	*14*	*58,547,167*	*0.16*	*1. 473 Caucasian* *2. 464 Caucasian*	*1.M* *2. F*	*17–65* *Post menopause*	*20 wks* *6 mths*	*3×/wk.* *120-170 min/wk*	*30–50 min* *120–170 min/wk*	*55–75% VO*_*2*_ *max* *+50%VO*_*2*_ *max*	*AA (−) n = 84*	*1.43* × *10-* ^5^	*Bouchard, 2011*
*NDN (75 kb) *Downstream of NDN*	*rs824205*	*15*	*21,559,164*	*0.15*	*1. 473 Caucasian* *2. 464 Caucasian*	*1.M* *2.F*	*17–65* *Post menopause*	*20 wks* *9 mths*	*3×/wk.* *120-170 min/wk*	*30–50 min* *120-170 m in/wk*	*55–75% VO*_*2*_ *max* *40–85%VO*_*2*_ *max*	*GG (−) n = 521*	*3.45* × *10~* ^5^ *0.05*	*Bouchard, 2011*
+DIS3L	rs1546570	15	64,382,829	NA	473 Caucasian	M&F	17–65	20 wks	3×/wk	30–50 min	55–75% VO_2_max	NA	2.3 × 10^−2^	Timmons, 2010
UNKL	rs3751894	16	1,426,876	NA	473 Caucasian	M	Young adults	1.6 wks 2. 12 wks	1. 4×/wk. 2. 3×/wk	1. 45 min 2. Progressive	1. 70% VO_2_max 2. Progressive	NA	NA	Timmons, 2010
IL32	rs13335	16	3,052,198	NA	473 Caucasian	M	Young adults	1.6 wks 2. 12 wks	1. 4×/wk. 2. 3×/wk	1. 45 min 2. Progressive	1. 70% VO_2_max 2. Progressive	NA	NA	Timmons, 2010
**#RPTOR**	**N/A**	**17**	**NA**	**NA**	**473 Caucasian**	**M&F**	**17–65**	**20 wks**	**3×/wk**	**30–50 min**	**55–75% VO**_**2**_ **max**	**NA**	**#89**	**Ghosh, 2013**
#VPS53	N/A	17	NA	NA	473 Caucasian	M&F	17–65	20 wks	3×/wk	30–50 min	55–75% VO_2_max	NA	#84	Ghosh, 2013
ACE	DI	17	58,919,622	NA	473 Caucasian	M&F	17–65	20 wks	3×/wk	30–50 min	55–75% VO_2_max	NA	NA	Timmons, 2010
SMTNL2	rs7217556	17	4,425,585	NA	41 Caucasian	M	Young adults	1.6 wks 2. 12 wks	1. 4×/wk. 2. 3×/wk	1. 45 min 2. Progressive	1. 70% VO_2_max 2. Progressive	NA	NA	Timmons, 2010
ZSWIM7	R21	17	15,825,286	NA	41 Caucasian	M	Young adults	1.6 wks 2. 12 wks	1. 4×/wk. 2. 3×/wk	1. 45 min 2. Progressive	1. 70% VO_2_max 2. Progressive	NA	NA	Timmons, 2010
ENOSF1	rs3786355	18	671,962	NA	41 Caucasian	M	Young adults	1.6 wks 2. 12 wks	1. 4×/wk. 2. 3×/wk	1. 45 min 2. Progressive	1. 70% VO_2_max 2. Progressive	NA	NA	Timmons, 2010
EMR4	rs7256163	19	6,909,134	0.31	473 Caucasian	M&F	17–65	20 wks	3×/wk	30–50 min	55–75% VO_2_max	NA	1.13 × 10-^4^	Bouchard, 2011
IER2	rs892020	19	13,8185	NA	41 Caucasian	M	Young adults	1.6 wks 2. 12 wks	1. 4×/wk. 2. 3×/wk	1. 45 min 2. Progressive	1. 70% VO_2_max 2. Progressive	NA	NA	Timmons, 2010
DNAJB1	rs4926222	19	14,488,050	NA	41 Caucasian	M	Young adults	1.6 wks 2. 12 wks	1. 4×/wk. 2. 3×/wk	1. 45 min 2. Progressive	1. 70% VO_2_max 2. Progressive	NA	NA	Timmons, 2010
g.63226200G > A	rs6090314	20	61,327,997	0.16	473 Caucasian	M&F	17–65	20 wks	3×/wk	30–50 min	55–75% VO_2_max	A (+)	1:6.48 × 10~^5^ 2:6.24 × 10^−5^	Bouchard, 2011 (1) Ghosh, 2013 (2)
^YTHDF1	rs6122403	20	NA	NA	473 Caucasian	M&F	17–65	20 wks	3×/wk	30–50 min	55–75% VO_2_max	NA	6.24 × 10^−5^	Ghosh, 2013
**^MACROD2**	**N/A**	**20**	**NA**	**NA**	**473 Caucasian**	**M&F**	**17–65**	**20 wks**	**3×/wk**	**30–50 min**	**55–75% VO**_**2**_ **max**	**NA**	**#86.6**	**Ghosh, 2013**
^HLS21	N/A	21	NA	NA	473 Caucasian	M&F	17–65	20 wks	3×/wk	30–50 min	55–75% VO_2_max	NA	#84.7	Ghosh, 2013
*MN1 (14 kb)	rs738353	22	26,460,072	0.35	473 Caucasian	M&F	17–65	20 wks	3×/wk	30–50 min	55–75% VO_2_max	A (+)	1.23 × 10–^4^	Bouchard, 2011
LOC731789	rs11015207	NA	NA	NA	473 Caucasian	M&F	17–65	20 wks	3×/wk	30–50 min	55–75% VO_2_max	NA	1.61 × 10^−4^	Ghosh, 2013

There were no other possible mediators (such as medications, health concerns) or other significant findings noted in the above three studies. Where possible, gene variants were annotated using the references sequence (GRCh37/hg19)

*Out of the 39 SNPs identified via GWAS, 21 (*) explained 49% of the VO_2_ max trainability variance (after regression analysis). The 15 most significant were then examined using data from the following studies: HERITAGE African-Americans, DREW study, STRRIDE study. The variants replicated are in italics

+11 SNPs from a regression analysis explained ~23% of the estimated VO_2_ max variance. 90% RNA expression remained unchanged by exercise training. (++) were found in study by Bouchard (2011) but weren’t included in the regression analysis because they weren’t considered significant at the 0.00015 level

^Top 20 GWAS associated genes based on second-best SNP-*P* values

#Candidate genes identified through CANDID software based on literature search; GWAS association data; sequence conversion & gene expression. This equates to a ‘final score’ rather than p-value. Bolded text indicates moderate-strong related biological mechanisms that influence VO_2_ max trainability

**(+) = significantly higher training response

(0) = no significant difference in training response between genotypes

(−) = significantly lower training response

**Table 4:** Predictor genes that may influence VO_2max_ training response

Number	Chromosome	Gene	Variant	Race	Genotype/expression and VO_2max_ training response (+/−/0)**	Author, Date (x = candidate gene study)
1	**1**	***AMPD1***	**rs17602729**	**Caucasian**	**TT and CT (−)**	**Thomaes, 2011 (x); Rico-Sanz, 2003 (x)**
2	1	***CAMTA1***	**rs884736**	**Caucasian** **African-American**	**AA (−)**	**Bouchard, 2011; Ghosh, 2013**
3	1	*ID3*	rs11574	Caucasian	TBC	Timmons, 2010
**4**	**1**	***RGS18***	**rs10921078**	**Caucasian** **African-American**	**GG (−)**	**Bouchard, 2011**
**5**	**1**	***RYR2***	**rs7531957**	**Caucasian**	**TBC**	**Bouchard, 2011; Ghosh, 2013**
6	1	*SLC45A1*	TBC	Caucasian	TBC	Ghosh, 2013
7	2	*ACVR1C*	TBC	Caucasian	TBC	Ghosh, 2013
8	2	*KCNF1*	rs2003298	Caucasian	TBC	Bouchard, 2011
9	2	*FLJ44450*	rs4952535	Caucasian	G (+)	Bouchard, 2011
10	2	*TTN*	rs10497520	Caucasian	TBC	Timmons, 2010
11	2	*NRP2*	rs3770991	Caucasian	TBC	Timmons, 2010
12	3	*KCNH8*	rs4973706	Caucasian	A (+)	Bouchard, 2011
**13**	**3**	***ZIC4***	**rs11715829**	**Caucasian**	**AA (−)**	**Bouchard, 2011**
14	3	*NLGN1*	rs2030398	Caucasian	A (+)	Bouchard, 2011
15	3	*ADCY5*	TBC	Caucasian	TBC	Ghosh, 2013
16	4	*CSN1S2B*	rs2272040	Caucasian	TBC	Bouchard, 2011
17	4	*LOC100289626*	rs2053896	Caucasian	A (+)	Bouchard, 2011
**18**	**4**	***ACSL1***	**rs6552828**	**Caucasian**	**AA (−)**	**Bouchard, 2011; Ghosh, 2013**
19	4	*SLED1*	rs6552828	Caucasian	TBC	Ghosh, 2013
20	4	*PRR27; C4orf40*	rs3775758	Caucasian	TBC	Ghosh, 2013
21	4	*TEC*	rs13117386	Caucasian	TBC	Ghosh, 2013
22	5	*NR3C1*	rs6190	Caucasian	GG (−)	Thomaes, 2011
23	5	*NLN*	TBC	Caucasian	TBC	Ghosh, 2013
24	5	*FABP6*	rs7734683	Caucasian	TBC	Ghosh, 2013
25	5	*TTC1*	rs2176830	Caucasian	TBC	Bouchard, 2011
26	6	*PPARD*	Exon 4 + 15 Exon 7 + 65	African-American	CC (−)	Hautala, 2007 (x)
27	6	*HCG22*	rs2517512	Caucasian	TBC	Ghosh, 2013
28	6	*HCG22*	rs2523849	Caucasian	TBC	Bouchard, 2011
29	6	*HCG22*	rs2523848	Caucasian	TBC	Bouchard, 2011
30	6	*HCG22*	rs2428514	Caucasian	TBC	Bouchard, 2011
31	6	*HCG22*	rs2517518	Caucasian	TBC	Bouchard, 2011
32	6	*HCG22*	rs2523840	Caucasian	TBC	Bouchard, 2011
33	6	*HCG22*	rs2517506	Caucasian	TBC	Bouchard, 2011
34	6	*PRDM1*	rs10499043	Caucasian	A (+)	Bouchard, 2011
35	6	*ENPP3*	rs10452621	Caucasian	A (+)	Bouchard, 2011
36	6	*SLC22A3*	rs2457571	Caucasian	Downregulated in high responders	Timmons, 2010
37	6	*TMEM181*	TBC	Caucasian	TBC	Ghosh, 2013
38	6	*PARK2*	TBC	Caucasian	TBC	Ghosh, 2013
39	6	*SNX14*	TBC	Caucasian	TBC	Ghosh, 2013
40	6	*BTBD9*	TBC	Caucasian	TBC	Ghosh, 2013
41	6	*KCNQ5*	TBC	Caucasian	TBC	Ghosh, 2013
42	7	*HDAC9*	rs3814991	Caucasian	TBC	Bouchard, 2011
43	7	*WBSCR17*	rs12538806	Caucasian	TBC	Bouchard, 2011
44	7	*WBSCR17*	rs13235325	Caucasian	TBC	Bouchard, 2011
45	7	*CPVL*	rs4257918	Caucasian	TBC	Timmons, 2010
46	7	*ITGB8*	rs10265149	Caucasian	TBC	Ghosh, 2013
47	7	*LHFPL3*	TBC	Caucasian	TBC	Ghosh, 2013
48	8	*DEPDC6*	rs7386139	Caucasian	TBC	Timmons, 2010
49	8	*PINX1*	TBC	Caucasian	TBC	Ghosh, 2013
50	9	*GRIN3A*	rs1535628	Caucasian	TBC	Bouchard, 2011
51	9	*GRIN3A*	rs959066	Caucasian	TBC	Bouchard, 2011
52	9	*C9orf27*	rs12115454	Caucasian	G (+)	Bouchard, 2011
53	9	*TTLL11*	rs7022103	Caucasian	TBC	Ghosh, 2013
54	9	*KCNT1*	TBC	Caucasian	TBC	Ghosh, 2013
55	10	*FAM238B; LOC731789*	rs11015207	Caucasian	TBC	Ghosh, 2013
56	10	*PRKG1*	TBC	Caucasian	TBC	Ghosh, 2013
57	10	*SVIL*	rs6481619	Caucasian	TBC	Timmons, 2010
58	10	*BTAF1*	rs2792022	Caucasian	TBC	Timmons, 2010
59	10	*CASC2*	rs1413184	Caucasian	TBC	Ghosh, 2013
60	11	*H19*	rs22551375	Caucasian	Upregulated in high responders	Timmons, 2010
61	11	*LOC100130460*	rs2198009	Caucasian	A (+)	Bouchard, 2011
62	11	*DBX1*	rs10500872	Caucasian	A (+)	Bouchard, 2011
**63**	**11**	***CD44***	**rs353625**	**Caucasian**	**TBC**	**Bouchard, 2011; Ghosh, 2013**
64	11	*CXCR5 (36 kb)*	rs4938561	Caucasian	TBC	Bouchard, 2011
65	11	*CXCR5 (24 kb)/BLR1*	rs7933007	Caucasian	TBC	Bouchard, 2011
66	11	*CD6*	rs175098	Caucasian	TBC	Ghosh, 2013
67	11	*SHANK2*	rs10751308	Caucasian	TBC	Ghosh, 2013
68	11	*GRIK4*	TBC	Caucasian	TBC	Ghosh, 2013
69	11	*CNTF*	rs1800169	Caucasian	AA (+)	Thomaes, 2011 (x)
70	11	*CAT*	-262C > T	Caucasian	TT (−)	Onkelinx, 2011 (x)
71	11	*GSTP1*	c.313A > G (rs1695)	Caucasian	GG & AG (+)	Zarebska, 2014 (x)
72	12	*FAM19A2*	rs2168452	Caucasian	TBC	Ghosh, 2013
73	12	*C12orf36*	rs12580476	Caucasian	TBC	Bouchard, 2011 Ghosh, 2013
74	13	*NALCN*	TBC	Caucasian	TBC	Ghosh, 2013
75	13	*MIPEP*	rs7324557	Caucasian	TBC	Timmons, 2010
76	13	*EEF1DP3*	rs2773968	Caucasian	TBC	Ghosh, 2013
77	13	*CLYBL*	NA	Caucasian	TBC	Ghosh, 2013
78	13	*Na + −K + −ATPase α2*	Alpha2 exon 1 Alpha2 exon 21–22	Caucasian	3.3/3.3 (−) 10.5/10.5 (+)	Rankinen, 2000a (x)
79	14	*HIF1A*	T + 140C	Caucasian (60+ years)	C/T (−)	Prior, 2003 (x)
80	14	*AKT1*	G205 T (RS1130214)	Caucasian men	GT & TT (+)	McKenzie, 2011 (x)
81	14	*TTC6*	rs12896790	Caucasian	C (+)	Bouchard, 2011
**82**	**14**	***DAAM1***	**rs1956197**	**Caucasian**	**AA (−)**	**Bouchard, 2011**
**83**	**15**	***NDN***	**rs824205**	**Caucasian**	**GG (−)**	**Bouchard, 2011**
84	15	*DIS3L*	Rs1546570	Caucasian	TBC	Timmons, 2010
**85**	**17**	***ACE***	**Intron 16**	**Caucasian**	**DD (+)** **II (+)**	**Rankinen, 2000b (x); Defoor, 2006 (x)**
86	17	*RPTOR*	NA	Caucasian	TBC	Ghosh, 2013
87	17	*VPS53*	NA	Caucasian	TBC	Ghosh, 2013
88	19	*ADGRE3P; EMR4*	rs7256163	Caucasian	TBC	Bouchard, 2011
**89**	**19**	***APOE***	**TBC**	**Chinese & unknown**	**E2/E3 (+)** **E2/E3 (+)** **E3/E4 (+)** **E3/E4 (+)** **E3/E3 (−)**	**Yu, 2014 (x); Thompson, 2004 (x)**
**90**	**19**	***CKM***	**Ncol**	**Caucasian**	**Homozygous 1170 bp (−); CKM locus (+/−)**	**Rivera, 1999(x); Rivera 1997 (x)**
**91**	**20**	***BIRC7 and YTHDF1***	**rs6090314**	**Caucasian**	**A (+)**	**Bouchard, 2011** **Ghosh, 2013**
92	20	*YTHDF1*	rs6122403	Caucasian	TBC	Ghosh, 2013
93	20	*MACROD2*	NA	Caucasian	TBC	Ghosh, 2013
94	21	*HLCS*	NA	Caucasian	TBC	Ghosh, 2013
95	22	*MN1*	rs738353	Caucasian	A (+)	Bouchard, 2011
96	Mitochondria	*ALAS2*	</=166 bp	Chinese	</=166 bp (+)	Xu, 2015 (x)
97	Mitochondria	*mtDNA*	TBC	Quebec, Tempe	mtDNA subunit 5 N5 (−)	Dionne, 1991 (x)

Where possible, gene variants were annotated using the references sequence (GRCh37/hg19)

Bolded = genes that have been replicated between or within studies

**(+) = high training response, (−) = low training response, (0) = neutral training response, *TBC* to be confirmed whether variant contributes to a high or low training response

## Results

**Fig. 1:**
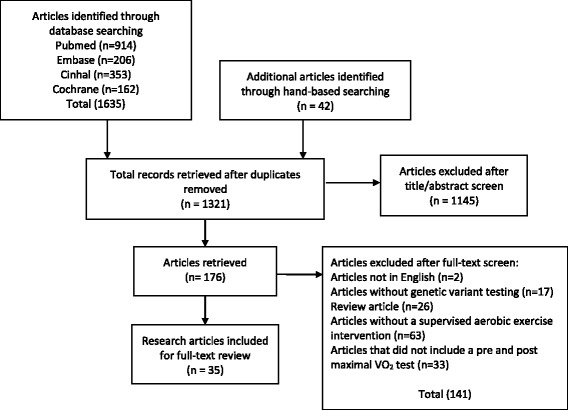
PRISMA flow chart of article selection process

Of the 1635 articles identified, 35 met the inclusion criteria (see Fig. 1). A summary of these articles is provided in Tables 1, 2 and 3. From the 35 articles, 97 genetic variants were identified as being significantly associated with VO_2max_ trainability (Table 4).

### Study characteristics

Across the studies DNA samples from 4212 individuals were used. Tissue sources were predominantly blood leucocytes, lymphoblastoid cell lines and buccal cells. Genotype was primarily identified through PCR-RFLP (polymerase chain reaction restriction fragment length polymorphism based analysis) for candidate genes and Illumina Human CV370-Quad Bead Chips for GWAS analysis (which can capture over 370,000 SNPs per participant).

Overall, 68% of participants in the reviewed studies were men, and ages ranged from 17 to 75 years. The average BMI of participants was 25.3 kg/m^2^ (SD 2.36)_._ Where detailed, DNA samples were taken from a variety of ethnicities, including Caucasian (74.5%), Asian (13.5%), African-American (7.5%), Hispanic (4.3%) and Native American (0.2%).

The 35 included articles described 15 cohorts, with three cohorts providing subject data for 19 articles (see Table 1 for details). Nine articles [20–28] used data from the HERITAGE study and five [29–33] reviewed Caucasian participant data from the Cardiac Rehabilitation and Genetics of Exercise Performance and Training Effect (CARAGENE) study. Five studies examined clinical data from 102 young male and apparently healthy police recruits in China [34–38]. The remaining samples came from independent clinical studies focusing on apparently healthy but sedentary adults from a variety of ethnicities including Caucasians, Asians, African-Americans, Native American and Hispanics [13, 39–53].

Most reviewed studies (*n* = 32) used a single-group longitudinal design. However, one study compared three groups using a longitudinal design [28]. One study used retrospective data from two Randomized Controlled Trials (RCT) [20]; and one was a double-blind study [39].

Twenty-eight studies examined a MICT intervention. Two studies examined protocols using High Intensity Interval Training (HIIT) [28, 40]. The 5 remaining studies trained participants by running at Ventilatory Threshold (VT) [34–38]. Training intensity was measured using a percentage of VO_2max_, Heart Rate Reserve (HRR), VT, Maximal Power (P_max_) or Maximum Heart Rate (HR_max_). Intensities varied between 50 and 85% VO_2max_, 95% -105% VT, 50–85% P_max_, 80–85% HRR and 50–80% HR_max_. Training volume varied between 20 to 90 min per session (2-4×/week). The period of interventions ranged from 4 weeks to 9 months. Training modalities consisted primarily of cycle ergometers and treadmills.

Only six studies incorporated a standardized diet prior to and during the intervention period [23, 41–45]. Three articles included strength training [20, 39, 47] and two studies included military training [39, 47] as the intervention.

### Genotyping findings

1. Candidate gene studies

The candidate gene association approach requires a prior hypothesis that the genetic polymorphisms of interest are causal variants or in strong linkage disequilibrium (LD) with a causal variant, and would be associated with a particular exercise-related phenotype at a significantly different rate than predicted by chance alone (may be higher or lower). This approach is effective in detecting genetic variants that are either directly causative, or belong to a shared haplotype that is causative [54]. Thirty-two candidate gene studies were based on the gene’s molecular function and possible association with VO_2max_ trainability (Table 2).

#### Genes associated with muscular subsystems

VO_2peak_ can be influenced by muscle efficiency and it has been hypothesized that genes encoding muscular subsystems may contribute to the genetic variability in VO_2peak_ training response [33]. Twelve genes and 21 genetic variants related to muscular phenotypes were investigated in 935 (76 female) cardiac patients from the CARAGENE study [33]. Three out of the 21 genetic variants were significantly associated (*p* < 0.05) with an increase in VO_2peak_ following 3 months of MICT (2–3 × 90-min sessions per week at 80% HR_max_; *p* < 0.05). These variants included *GR*:c.68 > A (G/A genotype, number of people with genotype; *n* = 55) in the glucocorticoid receptor gene (*GR*; rs6190), *CNTF*:c.115-6G > A (AA genotype, *n* = 21) in the ciliary neurotrophic factor gene (*CNTF*; rs1800169) and the *AMPD1*:c.133C wild type (CC genotype, *n* = 652) of the adenosine monophosphate deaminase gene (*AMPD1*; rs17602729). Furthermore, a larger change in relative VO_2peak_ was reported in patients with a greater number of these variants described (Area Under the Curve (AUC): 0.63; 95% Confidence Interval (CI): 0.56–0.7; *p* < 0.01). More specifically, those with a gene predictor score (GPS) of one or less positive response alleles had an average increase in VO_2peak_ of 16.7%. Those with four or more positive response alleles had an average increase of 25%, with each positive response allele contributing approximately 1% (13.5 mL/min) to the increase in VO_2peak_.

Caucasians aged between 17 and 65 years from the HERITAGE study who were homozygous (TT genotype) for the *AMPD1*:c.133C > T (p.(Gln45*)) (rs17602729) variant (*n* = 6), had a lower VO_2max_ training response (<121 mL/min; *p* = 0.006), compared to the CT and CC genotypes (*n* = 497) following 20 weeks of MICT (3 × 50 min per week at 55–75% HR_max_) [46].

The serine/threonine protein kinase 1 (*AKT1*) gene has been linked to growth and skeletal muscle differentiation [44]. In a study of 109 Caucasians (50–75 years old), men (*n* = 22) with the *AKT1*:c.-350G > T (rs1130214) variant (TT/GT genotype) significantly increased their VO_2max_ compared to men (*n* = 29) with the GG genotype (fold increase of 1.2 ± 0.02 vs 1.1 ± 0.02, *p* = 0.037) following 24 weeks of MICT (3 × 20–40 min per week at 50–75% HRR) [44].

The glutathione S-transferase P1 (*GSTP1*) c.313A > G variant has been associated with an impaired ability to remove excess reactive oxygen species. This is hypothesised to increase the exercise training response by better activation of cell signalling pathways resulting in positive muscle adaptations [45]. While investigating 62 Polish females’ (19–24 years-old) response to 12 weeks of MICT (3 × 60 min per week at 50–75% HR_max_), participants (*n* = 30) with the *GSTP1*:c.313A > G (GG + GA genotype) demonstrated a 2 mL/kg/min greater improvement in VO_2max_ compared to AA genotypes (*n* = 5) following training (absolute *p* = 0.029, relative *p* = 0.026, effect size = 0.06) [45].

#### Genes associated with electrolyte balance

The electrogenic transmembrane ATPase (*NA+/K + −ATPase*) gene may contribute to VO_2max_ trainability by affecting the electrolyte balance and membrane excitability in working muscles [24]. Examining Caucasian data from the HERITAGE study, it was found that those homozygous for a recurrent 3.3-kb deletion in the exon 1 of the *ATP1A2* gene (*n* = 5) had a 41% (45 mL/min) lower training response compared to heterozygotes (*n* = 87) [24]. This exon encodes on part (alpha-2-subunit) of the *Na+/K + ATPase* protein. This genotype also had a 48% (197 mL/min) lower VO_2max_ training response than homozygotes (*n* = 380) for a repeated 8.8-kb in the exon 1 of the *ATP1A2* gene following 20 weeks of MICT (*p* = 0.018) [24]. VO_2max_ gains were 29% (130 mL/min) and 39% (160 mL/min) greater in offspring homozygous for a 10.5-kb deletion in exon 21–22 (*n* = 14) compared to heterozygotes (*n* = 93) and homozygotes (*n* = 187) respectively (*p* = 0.017) [24].

The angiotensin-converting enzyme (*ACE*) gene contributes to blood pressure, fluid and salt balance [55]. Elite endurance athletes are more likely to have the Insertion (I) allele [56] which relates to lower *ACE* activity and reduced blood pressure response during exercise, whereas sprint/power athletes are more likely to have the Deletion (D) allele and the DD genotype [57] and subsequently higher *ACE* activity. Caucasians from the CARAGENE study with the homozygous II genotype (frequency of 0.23 and 0.18 for men and women respectively) had a 2.1% greater VO_2max_ training response (*p* = 0.047) compared to the DD genotype (frequency of 0.3 and 0.36 for men and women respectively) [31]. When eliminating those on *ACE* inhibitors, the improvement increased by 3% (*p* = 0.013) [31]. On the other hand, VO_2max_ trainability was 14–38% greater (*p* = 0.042) in HERITAGE Caucasian offspring with the DD genotype (*n* = 81) [25]. Three studies found no association with *ACE* or angiotensinogen genetic variants and VO_2max_ training response in 53 Caucasians (average age 19 years) following 12 weeks of military training [47]; 147 multi-ethnic 19–24 year-old adults following 8 weeks of military training [39]; and 83 Brazilian policemen (average age 26 years) following 17 weeks of MICT (3 × 60 min per week at 50–85% VO_2peak_) [48].

#### Genes associated with lipid metabolism

Genotypes of the perilipin (*PLIN1*) gene may influence training response via intracellular lipolysis and energy production [43]. In 101 Caucasians (50–75 years old), there were no significant differences between carriers and non-carriers of the *PLIN1*:c.504 T > A variant (rs1052700) after 24 weeks of MICT (20–40 min, 3 × per week) [43].

The peroxisome proliferator activated receptor delta (*PPARD*) gene affects fatty acid oxidation and energy production [22]. African-Americans (*n* = 19) from the HERITAGE study with the *PPARD* exon 4 + 15 (CC genotype) had a significantly lower VO_2max_ training response (> 50 mL/min lower; *p* = 0.028) and power output (> 15 W lower; *p* = 0.005) compared to the C/T and TT genotypes (*n* = 230) [22].

Apolipoprotein E (*APOE*) variants affect the level of lipids in the blood, cell lipid uptake and endothelial vascular dilation [23]. *APOE* has 3 common alleles: E2 (TT/TT), E3 (TT/CC), E4 (CC/CC) at two SNPs (rs429358, rs7412), which can create six possible genotypes (E2/E2, E3/E3, E4/E4, E2/E3, E2/E4, E3/E4) [58]. The *APOE* E4 allele has been associated with Alzheimer’s disease [59], higher levels of low density cholesterol (LDL-C) and a greater risk of coronary heart disease compared to E3 (wild-type) and E2 carriers [23]. Chinese men (18–40 years) with the *APOE* E2/E3 (*n* = 20) and E3/E4 (*n* = 31) genotypes had a significantly higher VO_2max_ training response (Odds Ratio (OR) = 0.68 (95% CI (0.04, 1.32); *p* = 0.04 and OR = 0.60 (95% CI (0.09, 1.11); *p* = 0.02 respectively) compared to other *APOE* genotypes following 6 months of progressive MICT (3 x per week at 60–85% VO_2_max) [13]. Similarly, Chinese women (18–40 years) with the *APOE* E2/E3 (*n* = 25) and E3/E4 (*n* = 29) genotypes had significantly higher VO_2max_ training responses compared to other *APOE* genotypes (OR = 0.62 (95% CI = 0.05, 1.18); *p* = 0.03 and OR = 0.62(95% CI = 0.09,1.15); *p* = 0.02 respectively) [13]. Men and women (ethnicity unknown) with the E3/E3 *APOE* genotype (*n* = 43) had an 8% lower training response compared to the E2/E3 (*n* = 40) and E3/E4 genotypes (*n* = 37) (*p* < 0.01, Bonferroni-corrected) following 6 months of MICT (4 × 50 min per week at 60–85% VO_2max_) [42]. However, there was no significant difference in the VO_2max_ training response between *APOE* genotypes in men and women from the HERITAGE study (*n* = 766) [23]. Similarly, in 51 males (40–80 years old, ethnicity not confirmed) there was no difference in VO_2max_ training response between genotypes [41].

#### Genes associated with oxidative phosphorylation and energy production

Mitochondrial DNA (*mtDNA*) encodes several enzyme subunits involved in oxidative phosphorylation, and may be a key factor in endurance and cardiorespiratory fitness [56]. Research of *mtDNA* variants in 41 inactive Japanese men (mean age 20.6) failed to find a significant difference in trainability after 8 weeks of MICT (3–4 × 60 min per week at 70% VO_2max_) [49]. On the contrary, 3 men (17–25 years) with the *mtDNA* variant in subunit 5 of ND5 had a lower VO_2max_ training response compared to other mtDNA variants (~ gain 0.22 L/min less, *p* < 0.05) following 12-weeks of MICT (3–5 × 45 min per week at 85%HRR_max_) [50].

The creatine kinase muscle (*CKM*) gene has been associated with reduced fatigue from increased adenosine triphosphate (ADP) production [26, 27]. Using data from the HERITAGE study, parents and offspring homozygote for the 1170 bp allele (*n* = 12) had a lower VO_2max_ training response (3 times and 1.5 times lower respectively; *p* < 0.05) compared to other *CKM* genotypes (*n* = 148). This explained 9 and 10% of the inter-individual variation in VO_2max_ change respectively [26]. A nominal genetic linkage was identified in siblings (*n* = 277) who shared two alleles (1170 base pairs or 985 + 185 base pairs) at the *CKM* locus identical by descent (IBD), with these siblings having similar changes in VO_2max_ compared to siblings with fewer alleles IBD (*p* = 0.04) [27]. In an earlier study focusing on muscle specific inherited variations, no association was found in 295 Caucasians (18–30 years old) between *CKM* or adenylate kinase (*AK1*) variants after a randomized control trial that included 15 weeks of endurance training versus maximal power contraction interval training [40]. Similarly, no association was found with the *CKM* gene and VO_2max_ trainability in 937 Caucasian patients with coronary artery disease following 3 months of MICT (2–3 × 90 min aerobic sessions per week at 80% HR_max_) [29].

Nuclear respiratory factor 1 *(NRF1*) and nuclear factor (erythroid-derived 2)-like 2 (*NFE2L2*) [36, 37], contribute to mitochondrial biogenesis and oxidative phosphorylation [60]. In a study involving 102 physically active Chinese male soldiers (average age 19 years), there was no association between *NRF1* and *NFE2L2* genotypes or haplotypes and VO_2max_ trainability after 18 weeks of 3 × 5000 m runs per week at 95–105% VT [36, 37].

#### Genes associated with oxygen delivery

Nitric oxide causes coronary and arterial vasodilation, contributing to oxygen delivery regulation [32]. Data from the CARAGENE study was used to investigate genes associated with nitric oxide bioavailability [32]. These included nitric oxide synthase 3 (*NOS3*), cytochrome b-245 alpha chain (*CYBA*, also known as *p22-PHOX*), glutathione peroxidase (*GPX1*), catalase (*CAT*), superoxide dismutase 3 (*SOD3*), vascular endothelial growth factor A (*VEGFA*), peroxisome proliferator-activated receptor alpha (*PPARα*) and peroxisome proliferator-activated receptor gamma coactivator-related 1 (*PPARC1*) [32]. Participants carrying the C allele of the *CAT*:c.262 T > C variant (*n* = 342) had up to 3.1% greater improvements in VO_2max_ training response compared to participants with the TT genotype (*n* = 521) following MICT (*f* = 3.6; *p* = 0.02). Participants with the *NOS3* 1.4 haplotype combinations (*n* = 36) had a 6.4% lower training response compared to the 3.3. haplotype combinations (*n* = 133) (*p* < 0.05). However, these associations were not significant after Bonferroni correction. No other associations were found with other genes or haplotypes related to nitric oxide availability and endothelial function [32]. Similarly, in a cohort of 80 Portuguese (20–35 years old) police recruits, there was no association between *NOS3* genotypes (−786 TT/TC/CC, 894 GT/TT/GG) and VO_2peak_ response following 18 weeks of 3 × 80-min per week of graded running training [59]. Additionally, no association was found with *PPARGC1* and VO_2max_ trainability in 102 Chinese male polices recruits following MICT [36].

The beta-2-adrenergic receptor (*ADBR2*) gene helps to support oxygen delivery to working muscles via the adrenergic receptors [30]. In participants from the CARAGENE study, there was no association found between *ADBR2* genotypes or haplotypes, and VO_2max_ trainability [30].

The hypoxia-inducible factor 1 alpha (HIF1A) gene is a transcriptional regulator that controls angiogenesis (blood vessel development) and metabolism by increasing the expression of hypoxia-induced genes, such as *VEGF* [52]. Caucasians 60 years and over with the *H1F1A*:c.1744C > T (rs11549465; C/T genotype; *n* = 37) had a significantly lower training response (0.3 mL/kg/min; *p* = 0.03) compared to those with the CC genotype (*n* = 64) following 24 weeks of MICT (3 × 20–40 min per week at 50–70% VO_2max_) [52].

The 5′-aminolevulinate synthase 2 (*ALAS2*) gene is highly expressed in erythroid cells and is imperative for hemoglobin and myoglobin synthesis [53]. Seventy-two Chinese participants (18–22 years old) allocated to one of 13 *ALAS2* genotypes with compound dinucleotide repeats lengths (157 bp −184 bp), were placed in a 4-week ‘HiHiLo’ training program (varying between low and high altitude training at 75% VO_2max_) [53]. Baseline hemoglobin levels and change in VO_2max_ with training was significantly higher in subjects (*n* = 25) with the dinucleotide repeats ≤ 166 bp (*p* < 0.05). No significant associations were found between VO_2max_ trainability and other genes related to oxygen transport and utilization genotypes in 102 young Chinese soldiers following 18 weeks of 3 × 5000 m runs per week [35, 37, 38]. These genes include mitochondrial transcription factor A (*TFAM*) [35] and hemoglobin-beta locus (*HBB*) [38].

2. Hypotheses free studies

Over the last decade, with the advent of technological advances allowing researchers to genotype millions of genetic variants (e.g. SNPs) in each individual, the investigation of the contribution of common variants to traits is now feasible. Unbiased and hypothesis-free genome wide association studies (GWAS) for exercise/health-related traits have emerged.

Three studies have used GWAS to identify genes associated with the VO_2max_ response to exercise training [20, 21 28]. These are outlined in Table 3.

The first investigated two clinical trials and data from the HERITAGE study [28]. RNA expression profiling and VO_2max_ testing was performed on 24 healthy and inactive Caucasian men (average age 24 years) before and after a 6-week training intervention (4 × 45-min cycling sessions per week at 70% VO_2max_). Muscle biopsies from the vastus lateralis were collected and the RNA expression of genes was correlated with changes in VO_2max_ by analysing oligonucleotide arrays. Pearson correlations were used to identify the relationships between the median logit normalised probe sets and the number of times they were selected. In the 24 subjects, using a median correlation cut-off greater than 0.3, 29 genes were selected greater than 22 out of 24 times. The sum of expression of these 29 genes were found to have a significant linear relationship with VO_2max_ change following endurance training (*r* ^2^ = 0.58, *p* < 0.00001). Across the group, VO_2max_ changes improved on average by 14% and ranged from −2.8% to 27.5% (*p* = 0.0001). More than 20% of the group had a response less than 5%. A gene set enrichment analysis found that the oxidative phosphorylation gene was upregulated (False Discovery Rate (FDR) = 1.1%), which was associated with an increased reliance on lipids during training (RER decreased on average by 10% post training, *p* < 0.0001). To identify if these predictor genes would be similar in a different sample, a 12-week blind study on 17 young and active Caucasian men was conducted. Training consisted of 1-day of testing, 2 sessions of interval training (3 × 3-min intervals at 40–85% P_max_) and 2 × 60–120-min cycle sessions (55–60% P_max_) each week. The 29 predictor genes were also significantly associated with VO_2max_ trainability in this group (*p* = 0.02). The haplotypes of these predictor genes were then genotyped using candidate genes identified from the HERITAGE study. Six genetic variants were associated with VO_2max_ trainability: *SMTNL2, DEPDC6, SLC22A3, METTL3, ID3* and *BTNL9* (*p* < 0.01 each). A stepwise regression model using 25 variants from the predictor set and 10 variants from the HERTIAGE study (Table 3) found that eleven SNPs (included in Table 4) contributed to 23% of the differences seen in residual VO_2 max_ gains, which correlated to approximately 50% of the genetic variability in VO_2max_ trainability (seven variants from the RNA predictor set and four from the HERITAGE project). Reciprocal RNA expression validation found that three of four HERITAGE candidate genes enhanced the original RNA transcript predictor model. Overall, more than 90% of gene expression did not change. However, *OCT3* was downregulated in high responders and *H19* was upregulated in low responders (FDR <5%). *BTNL9, KLF4* and *SMTNL2* also had small but inconsistent changes in expression (i.e. dissimilar in high vs low responders) (FDR < 5%).

A GWAS examining 324,611 variants from the HERITAGE study was completed to identify possible predictor genes associated with VO_2peak_ [20]. Based on single-variant analysis, 39 variants (Table 3) were associated with gains in VO_2peak_ although none of these achieved genome-wide or suggestive significance (*p* = 1.5 × 10^−4^) [19]. The strongest predictor for training response was found in the Acyl-CoA synthetase long-chain family member 1 (*ACSL1*) gene (4:g.185725416A > G; rs6552828) which accounted for 7% of the training response (*p* = 1.31 × 10^−6^). After a stepwise multiple regression analysis of the thirty-nine variants, 21 were suggested to account for (or at least contribute to) 49% of the variance in VO_2max_ trainability (included in Table 4; *p* < 0.05). The strongest predictors were found in SNPs associated with: PR domain-containing protein 1 (*PRDM1*); glutamate receptor, ionotropic, N-methyl-D-aspartate 3A (*GRIN3A*); N-methyl-D-aspartate receptor (*NMDA*); potassium voltage-gated channel subfamily H member 8 (*KCNH8*); zinc finger protein of cerebellum 4 (*ZIC4*); and, *ACSL1*. An unweighted ‘predictor score’ based on contribution to VO_2max_ of these 21 variants was created. A score of ‘0’ represented homozygote for the low-response variant; ‘1’ represented heterozygous and ‘2’ represented homozygous for the high-response allele. Individuals with a score equal to or less than 9 (*n* = 36) had an average VO_2max_ score improvement of 221 mL O_2_/min. Alternatively, those (*n* = 52) with a score equal to or greater than 19 had an average VO_2max_ increase of 604 mL/min.

The 15 most significant variants were tested for replication in a sample of African-Americans from the HERITAGE study, women in the Dose Response to Exercise (DREW) study (*n* = 112), and the men and women in the Study of a Targeted Risk Reduction Intervention through Defined Exercises (STRRIDE) (*n* = 183) [20]. Variants in the *NDN* (15:g.24008071 T > C; rs824205) and *DAAM1* (14:g.59477414C > T; rs1956197) were replicated in the DREW study, the *Z1C4* (3:g.146957166 T > C T; rs11715829) variant was replicated in the STRRIDE study and *CAMTA1* (7:g.7015105 T > C; rs884736) and *RGS18* (1:g.192059022G > A; rs10921078) variants were replicated in African-Americans from the HERITAGE study. Four variants in the genes supervillin (*SVIL*), neuropillin 2 *(NRP2*), titin (*TTN*) and carbozypeptidase (*CPVL*) identified by Timmons et al. [28] were also found by Bouchard et al. [20], however, at a significance of 0.008, these variants were not included in the multi-variate regression analysis.

Using the HERITAGE cohort, an extended analysis was performed, with 2.5 million variants analysed [21]. To reduce bias associated with outlier variants, the second most significant variant *p*-value was used to determine genotype and changes in VO_2max_. Even with an extended analysis, the *ACSL1* gene was shown to have the most significant variant (4:g.185725416A > G; rs6552828), which confirmed findings by Bouchard et al. [20], whom identified the most significant variant at each gene (Table 3). The following genes and their variants were also replicated in both studies: *CAMTA1* (rs884736), *RYR2* (rs7531957), g.63226200G > A (rs6090314), *C12orf36* (rs12580476) and *CD44* (rs353625) [20, 21].

The gene prioritisation tool ‘CANDID’ was then used to rank candidate genes for changes in VO_2max_ [21]. This was done via: 1) a weighted analysis based on variant gene expression in targeted tissues; 2) GWAS p-value change in VO_2max_; 3) literature related to candidate genes; and 4) ‘cross species sequence conservation’ [21]. The top-ranking candidate genes from the GWAS and CANDID tool (Table 1) were then investigated for possible biological mechanisms and changes in VO_2max_. As a result, variants were allocated into four groups: 1) broad effects on exercise-related processes (such as the electron transport chain, physical fitness, skeletal development and other cardiorespiratory markers); 2) moderately strong scores against selective exercise-related processes; 3) high and low scores across several exercise-related processes; 4) low scores across all exercise-related processes.

Variants and their involvement in pathways related to changes in VO_2max_ response were then examined [21]. Out of the sixteen pathways found, variants related to pantothenate and co-enzyme A (CoA) biosynthesis, *PPAR* gene signalling and immune function signalling had the highest level of ‘burden’ (variants contributing to trainability). The variants related to long-chain fatty acid transport (including *ACSL1*) and fatty acid oxidation strongly influence VO_2max_ training response via lipid metabolism process and the tricarboxylic acid cycle, both of which affect the availability of adenosine triphosphate and subsequently training response.

### Predictor genes

Out of the 35 articles analysed (candidate genes and GWAS studies), 97 predictor genes were identified as possible contributors to VO_2max_ trainability (Table 4). These genes were based on what authors deemed significant, or the most significant, for their particular study. Thirteen of these predictor genes were replicated between at least two studies (bolded in Table 4). The traits for VO_2max_ trainability (e.g. which genotype was related to the training effect and whether it was a low or high responding genotype) was not outlined for each variant and hence this will require confirmation in future studies.

## Discussion

This systematic review aimed to summarize genetic variants that have been identified as influencing VO_2max_ trainability. We have reviewed 35 studies that have reported 97 genes associated with an exercise training-induced improvement in VO_2max_. It has been estimated that VO_2max_ trainability has a significant heritable component of around 50% [39].

There were several studies that identified the same variant, including: the lipid-related *ACSL1*:c.-32-716 T > C (rs6552828) [20, 21] and skeletal muscle-related *AMPD1*:c.133C > T [33, 46]; intra-cellular calcium regulator *RYR2*:c.6166 + 552 T > G; cellular function-related *CD44* (rs3653625), transcriptional activator *CAMTA1* (rs884736), non-coding *C12orf36* (rs12580476) and apoptotic regulator *20:g.63226200G > A (*rs6090314) [20, 21]. Additionally, Bouchard et al. [20] were able to replicate the variants in genes from the HERITAGE study, including: growth suppressor *NDN*, cell cortex function-related *DAAM1*, development-related *Z1C4* and signal transduction inhibitor *RGS18*. Numerous identified variants were found in pathways that contribute to training response (e.g. calcium signaling, immune function, angiogenesis, mitochondrial biogenesis) with pathways and associated SNPs possibly influencing each other and overall trainability [21]. Several articles found conflicting results with electrolyte balance, lipid production and energy production genes *ACE* [25, 31, 47, 48], *APOE* [13, 23, 41, 42], *mtDNA* [49, 50] and *CKMM* variants respectively [26, 27, 29, 40]. All other ‘predictor genes’ identified are yet to be replicated.

While most of the articles examined in this review have focused on one or a few candidate genes/markers (*n* = 32), it is noted that exercise-related phenotypes are complex traits and are polygenic (i.e. influenced by many genes working together) with each genetic variant likely to be contributing a small percentage (typically less than 1%) to the overall change in VO_2max_ [33, 39, 61]. Thus relying on one variant as a predictor is misguided; rather it has been suggested that a gene predictor score (GPS) based on numerous variants has a greater probability to determine higher and lower responders for VO_2max_ trainability. For example, a score of ‘0’ represents a homozygote for a low-response variant; ‘1’ represents heterozygous and ‘2’ represents homozygous for a high-response variant [20]. A higher score indicates a greater possible VO_2max_ training response (and vice versa). A similar model has been suggested in elite athletes aiming to determine the probability of an individual with a theoretically ‘optimal’ polygenic profile for endurance sports. The ‘optimal’ profile using a so-called ‘total genotype score’ (TGS, ranging from 0 to 100, with ‘0’ and ‘100’ being the worst and best genotype combinations, respectively) was quantified from a simple algorithm resulting from the combination of candidate polymorphisms [62, 63].

These predictor genes, along with muscle RNA and protein expression data provide a sound platform to further explore the cellular mechanisms underlying VO_2max_ trainability. Further research will need to consider several limitations identified from the literature to-date. For example, the lack of replication found between articles and conflicting results with certain variants, may be a result of several main limitations (typically in study design). Firstly, most of the articles used a hypotheses-driven candidate gene approach (*n* = 32), several articles used retrospective data from similar cohorts (*n* = 19), and many lacked a control group and randomization (*n* = 31). While it is understandable that in the past, high-throughput SNP microarray or gene sequencing technology was not available to use, by looking at one or only a few gene variants (whereas it is estimated that the human genome consists of about 40 million common gene variants) it is almost impossible to generate meaningful information. Similarly, a lack of control group makes it challenging to distinguish between individual response to an intervention and within-subject random variation [64]. Secondly, most of the exercise training studies involve a relatively small number of participants (typically *n* = 20 to 30; with the exception of the HERITAGE and CARAGENE studies), which results in lack of statistical power when associating genotype with a phenotype. Many of the studies also failed to include a robust significance criterion (*p* < 0.05 occurs approximately 10^6^ times in the genome by chance). Thirdly, a lack of racial diversity (74.5% Caucasian) further reduces the power of variants detected. Finally, many of the training studies were not tightly controlled in terms of nutrition, participant baseline data (study entry), physical activity status and other lifestyle factors.

Future research needs to consider epigenetic variation of gene activity that can occur in reaction to external factors, such as additional physical activity, drugs, diet and environmental toxins [61, 65]. Such epigenetic modifications can affect all adaptions to exercise training [10]. For example, in addition to nutrition and baseline physical activity status, there were many other differences in subjects between articles not taken into consideration including: age, training duration and volume (MICT vs. HIIT), body weight, body fat percentage, medications, clinical versus healthy populations; sleep, psychological status and the gut microbiome. Together, these are potential epigenetic modifiers (e.g. DNA methylation and histone acetylation) that can influence gene expression, molecular function and thereby influence VO_2max_ training response [61, 66]. Whether genes or epigenetic modifiers play a larger percentage role in adaptive variability in a specific situation requires further exploration.

To address these limitations, larger-scale studies are required to ascertain if the 97 predictor genes identified from this review are similar in various cohorts (e.g. several ethnicities, ages, gender). The Athlome Project Consortium, which includes the Gene SMART study, is an example of a current larger-scale investigation examining ‘omic markers’ of training response, elite performance and injury rates/predisposition in variety of populations [67]. Ideally, future studies will complement and expand on this research, and consider alternative forms of exercise training intensity and volume, lifestyle factors, general health, diet, medications and health history when implementing interventions and analyzing data.

Furthermore, the role of the gut microbiome, and its influence on metabolism and physiology, needs to be explored. For example, gut microbiota (which has its own genome) can interact with the tissue cellular environment to regulate gene expression [61]. Poor diet, stress, illness, the use of antibiotics, environmental toxins and poor lifestyle choices can increase inflammation within the gut, causing dysbiosis; this appears to contribute to chronic diseases and other illnesses, irrespective of genotype, age and gender [68, 69]. Interestingly, VO_2max_ was recently shown to be related to gut microbial diversity in a human cross-sectional study [70], suggesting a link between VO_2max_ and gut microbes. Pre- and probiotics, resistant starch and a Mediterranean diet (dietary diversification) can alter the gut microbiome [68]. Investigating how the gut and human genome interact to positively influence VO_2max_ is warranted.

With these points in mind, the analysis of stool samples, in addition to incorporating epigenetic, transcription and proteomic analysis, may help to identify the best aerobic training or lifestyle intervention to upregulate or downregulate certain genes, signaling pathways and molecular responses required for a greater VO_2max_ training response. Implementing tightly-controlled studies examining various mediators (training intervention, diet, lifestyle) and molecular biomarkers across various populations will help to capture accurate information related to ideal traits for VO_2max_ trainability.

## Conclusion

In total, 97 genes that predicted VO_2max_ trainability were identified. Phenotype is dependent on several of these genotypes/variants, which may contribute to approximately 50% of an individual’s VO_2max_ trainability. Higher responders to exercise training have more positive response alleles (greater gene predictor score) than lower responders. Whilst these findings are exciting, further randomized-controlled research with larger and diverse cohorts are needed. Additional exploration is required to identify genetic variants and the mediators (training intensity and volume, diet, drugs, other lifestyle factors) that can potentially affect gene expression, molecular function and training response. Findings from this review and future research may assist clinicians to provide precision evidence-based medicine centered on phenotype, contributing to the fight against chronic disease.

### Pubmed, embase, cinahl and cochrane search terms

#### Pubmed search

gene*[ti] OR allele [tiab] OR SNP [tiab] OR genetic profiling[tiab] OR genetic variant*[tiab] OR Genomic predictor*[tiab] OR polymorphism[tiab] OR heritability[tiab] AND (exercise training [tiab] OR VO2peak[tiab] OR ‘cardiorespiratory fitness’[tiab] OR ‘maximal/maximum VO2peak’[tiab] OR maximal/maximum VO2max’[tiab] OR maximal oxygen consumption’[tiab]OR peak oxygen uptake’[tiab] OR interval exercise’[tiab] OR ‘high/low intensity exercise’[tiab] OR peak fitness [tiab] OR endurance*[tiab] OR physical fitness[tiab] OR cardiorespiratory fitness[tiab] OR endurance training [tiab] OR cardiovascular fitness[tiab] OR VO2max[tiab] OR aerobic power[tiab] OR aerobic fitness[tiab] OR exercise capacity[tiab] OR exercise training response[tiab] OR response to exercise training[tiab]) NOT animal*.

#### Embase

gene:ab,ti OR allele:ab,ti OR snp:ab,ti OR ‘genetic profiling’:ab,ti OR ‘genetic variant’:ab,ti OR ‘genomic predictor’:ab,ti OR heritability:ab,ti AND (vo2peak:ab,ti OR vo2max:ab,ti OR ‘cardiovascular fitness’:ab,ti OR ‘cardiorespiratory fitness’:ab,ti OR ‘aerobic power’:ab,ti OR ‘aerobic fitness’:ab,ti OR ‘exercise training response’:ab,ti OR ‘physical fitness’:ab,ti).

#### Cinahl

(genes OR ‘genetic variant’ OR ‘Genomic predictor’ OR polymorphism OR ‘genetic profiling’ OR ‘single nucleotide polymorphisms’ OR ‘SNPs’ heritability) AND (‘trainability’ OR’ cardiovascular fitness’ OR ‘interval exercise’ OR ‘maximum O2’ OR maximal oxygen consumption’ OR ‘peak oxygen consumption’ OR maximal aerobic capacity’ OR ‘high/low intensity exercise’ OR ‘cardiorespiratory fitness’ OR ‘aerobic power’ OR ‘response to exercise training’ OR ‘exercise capacity’ OR ‘VO2max’ OR ‘VO2peak’ OR endurance).

#### Cochrane database for systematic reviews

(genes OR ‘genetic variant’ OR ‘Genomic predictor’ OR polymorphism OR ‘genetic profiling’ OR ‘single nucleotide polymorphisms’ OR ‘SNPs’ OR heritability) AND (‘trainability’ OR’ cardiovascular fitness’ OR ‘interval exercise’ OR ‘maximum O2’ OR maximal oxygen consumption’ OR ‘peak oxygen consumption’ OR maximal aerobic capacity’ OR ‘high/low intensity exercise’ OR ‘cardiorespiratory fitness’ OR ‘aerobic power’ OR ‘response to exercise training’ OR ‘exercise capacity’ OR ‘VO2max’ OR ‘VO2peak’ OR endurance).

#### Cochrane central register of controlled trial

(genes OR ‘genetic variant’ OR ‘Genomic predictor’ OR polymorphism OR ‘genetic profiling’ OR ‘single nucleotide polymorphisms’ OR ‘SNPs’ heritability) AND (‘trainability’ OR’ cardiovascular fitness’ OR ‘cardiorespiratory fitness’ OR ‘interval exercise’ OR ‘maximum O2’ OR maximal oxygen consumption’ OR ‘peak oxygen consumption’ OR maximal aerobic capacity’ OR ‘high/low intensity exercise’ OR ‘aerobic power’ OR ‘response to exercise training’ OR ‘exercise capacity’ OR ‘VO2max’ OR ‘VO2peak’ OR endurance).
